# Estimation of Synaptic Conductances in Presence of Nonlinear Effects Caused by Subthreshold Ionic Currents

**DOI:** 10.3389/fncom.2017.00069

**Published:** 2017-07-25

**Authors:** Catalina Vich, Rune W. Berg, Antoni Guillamon, Susanne Ditlevsen

**Affiliations:** ^1^Departament de Matemàtiques i Informàtica, Universitat de les Illes Balears Palma, Spain; ^2^Center for Neuroscience, University of Copenhagen Copenhagen, Denmark; ^3^Departament de Matemàtiques, Universitat Politècnica de Catalunya Barcelona, Spain; ^4^Department of Mathematical Sciences, University of Copenhagen Copenhagen, Denmark

**Keywords:** synaptic inhibition and excitation, quadratic integrate-and-fire model, Ornstein-Uhlenbeck process, oversampling method, spinal motoneurons, intracellular recordings of membrane potentials, maximum likelihood estimation, intrinsic currents

## Abstract

Subthreshold fluctuations in neuronal membrane potential traces contain nonlinear components, and employing nonlinear models might improve the statistical inference. We propose a new strategy to estimate synaptic conductances, which has been tested using *in silico* data and applied to *in vivo* recordings. The model is constructed to capture the nonlinearities caused by subthreshold activated currents, and the estimation procedure can discern between excitatory and inhibitory conductances using only one membrane potential trace. More precisely, we perform second order approximations of biophysical models to capture the subthreshold nonlinearities, resulting in quadratic integrate-and-fire models, and apply approximate maximum likelihood estimation where we only suppose that conductances are stationary in a 50–100 ms time window. The results show an improvement compared to existent procedures for the models tested here.

## 1. Introduction

Unveiling the information that a neuron receives from other neurons and distinguishing between excitatory and inhibitory inputs is an important task in neuroscience as it provides valuable information on local connectivity and brain operating conditions. From an experimental point of view, this is difficult due to the diversity of synaptic inputs and their unattainable conductances. Therefore, inverse methods are sought to retrieve the dynamics of mean synaptic conductances from measurements of the membrane potential of the neuron. Different statistical tools have been proposed to solve this problem in the literature, but they have individual drawbacks. First, it is desirable that such methods do not rely on repeated trials since it is difficult to assume exactly repeated synaptic input from trial to trial. Therefore, estimation of excitatory and inhibitory conductances from single time courses of the membrane potential is preferable. Second, estimation methods should be based on few assumptions in order to be applied in as general conditions as possible, and therefore, the underlying mathematical models have to be as nonspecific as possible. Finally, one has to take into account the presence of noise as well as potential nonlinearity in the experimental data.

As far as we know, no complete solutions to these constraints are yet available. On one hand, many existing methods require repeated trials of recordings of the membrane potential (see Monier et al., 2008 for a review). On the other hand, almost all methods in the literature are based on linear models (see e.g., Borg-Graham et al., 1998; Anderson et al., 2000; Wehr and Zador, 2003; Rudolph et al., 2004; Pospischil et al., 2009; Bédard et al., 2012; Berg and Ditlevsen, 2013; Kobayashi et al., 2016; Yasar et al., 2016). Noise has been taken into account through stochastic linear processes (Rudolph et al., 2004; Pospischil et al., 2009; Paninski et al., 2012; Berg and Ditlevsen, 2013, among others) or sophisticated filtering techniques (e.g., Lankarany et al., 2013; Closas, 2014; Ditlevsen and Samson, 2014; Lankarany et al., 2016), whereas some methods are restricted to deterministic models (e.g., Bédard et al., 2012; Vich and Guillamon, 2015).

It has been reported that linear models provide poor estimates in spiking regimes (Guillamon et al., 2006), so most of the above methods only apply to data from subthreshold activity. Linear models have provided good approximations of subthreshold neural activity (Jolivet et al., 2004; Kobayashi et al., 2009; Berg and Ditlevsen, 2013). However, if ionic currents inducing nonlinearities in the subthreshold regime are active (such as situations where resonant and amplifying currents coexist, see Rotstein, 2015, or in presence of the *I*_*T*_ current, see Destexhe and Babloyantz, 1993), linear models can also lead to poor estimates in subthreshold regimes (Vich and Guillamon, 2015). Thus, taking a linear model as a generic model does not seem a valid strategy in all situations; even with some data treatment, such as filtering the observed trace, the transformed dynamics cannot always be assumed to follow a linear model. A new procedure to estimate conductances taking into account possible subthreshold activated currents was introduced in Vich and Guillamon (2015). It is based on a quadratization of a deterministic model, and significantly improves estimates when compared to those obtained by the common linear methods. However, the method does not incorporate noise and, moreover, it requires the use of voltage traces from two trials.

The obstacles discussed above motivated us to seek for estimation approaches using single-trial data and stochastic models incorporating nonlinear effects. The main goal of this paper is to estimate synaptic conductances based on a quadratization of a stochastic model. The approach is based on a combination of the methods from Berg and Ditlevsen (2013) and Vich and Guillamon (2015) in order to capture both subthreshold noise and nonlinearities in the experimental data. One method presented in Berg and Ditlevsen (2013), which we refer to as the *OU method*, since it is based on an Ornstein-Uhlenbeck process, is effective in dealing with noise using single trial voltage traces. Thus, we extend the OU method by adding a quadratic term to the underlying model; more specifically, by considering a stochastic version of the *quadratic integrate-and-fire* (QIF) model (see Latham et al., 2000 and Hansel and Mato, 2001). In Berg and Ditlevsen (2013) both maximum likelihood estimation and estimation of the membrane time constant via the autocorrelation function are proposed. Here we explore approximate maximum likelihood estimation, since the exact likelihood and autocorrelation function are not available for the QIF model. For voltage values close to the spiking threshold, the QIF model is a good candidate to approximate subthreshold dynamics of conductance-based models since it can reproduce the bifurcation structure of the biophysical models (see Ermentrout and Kopell, 1986), even in the presence of active nonlinear currents. In this regime, the linear methods can be inaccurate.

We apply the statistical model to estimate synaptic conductances from both simulated and experimental data. Simulated data are obtained from two different neuron models using prescribed synaptic inputs. Experimental data come from intracellular recordings in current-clamp mode of spinal motoneurons of red-eared turtles, and have been analyzed elsewhere (Berg et al., 2007; Jahn et al., 2011; Berg and Ditlevsen, 2013). Given a membrane potential trace, we fit the data to the reference QIF model and estimate the time course of the conductances by means of an approximated maximum likelihood procedure. In the case of simulated data, we compare the estimated conductances with the true ones to evaluate the performance of the procedure, as well as compare the method with two previously proposed methods, which only need a single voltage trace: the *oversampling method* (Bédard et al., 2012), based on a deterministic approach, and the OU method (Berg and Ditlevsen, 2013) mentioned above.

The paper is structured as follows: in Section 2, the estimation procedure, the neuron models used to generate the *in silico* data, and the experimental setup for the *in vivo* intracellular voltage traces are described. In Section 3, we show the results obtained when the estimation procedure is applied to both *in silico* and *in vivo* voltage traces. We also compare the estimates to those obtained by the OU and the oversampling methods based on single-trial recordings. We discuss the results in Section 4. We include three appendices devoted to the more technical details of the models and the procedure.

## 2. Methods

In this Section we describe the new procedure to estimate the time course of the synaptic conductances. We also briefly describe the OU and the oversampling methods from the literature, with which we will later compare. We generate *in silico* voltage traces from computational neuron models using prescribed synaptic inputs generated from Ornstein-Uhlenbeck processes with sinusoidal drift, or use *in vivo* data of intracellular recordings of a spinal motoneuron subjected to rhythmic synaptic bombardments from the surrounding network. Finally, we explain how to embed a given biophysical model or an experimental data trace into the QIF model.

### 2.1. New estimation procedure: QIF method

To capture nonlinearities in the subthreshold regime, we use the QIF model as a base model for the estimation procedure. It is given by a single equation for the membrane potential *V*(*t*),

(1) $$\begin{array}{l} C\frac{dV(t)}{dt}=\alpha {\left(V(t)-V_{T}\right)}^{2}-I_{T}-g_{E}(t)\left(V(t)-V_{E}\right)\\ \;-\;g_{I}(t)\left(V(t)-V_{I}\right)+I_{app}+\eta (t), \end{array}$$

where *C* is the total capacitance, *g*_*E*_(*t*) and *g*_*I*_(*t*) are the time-varying excitation and inhibition conductances, *V*_*E*_ and *V*_*I*_ are their respective reversal potentials, η(*t*) is a zero mean noise process taking into account the random arrivals of synaptic input, and *I*_*app*_ is the applied current imposed by the experimenter. Furthermore, specific to the quadratic model, *I*_*T*_ is the largest input current at which the neuron does not spike in the absence of synaptic input, applied current and noise, *V*_*T*_ denotes the corresponding voltage of the *V* − *I* curve at *I*_*T*_, and α = *g*_*L*_/(2Δ_*T*_), where *g*_*L*_ is the leak conductance and Δ_*T*_ is the spike slope factor at *I*_*T*_, which corresponds to the inverse of the curvature of the *V* − *I* curve at (*I*_*T*_, *V*_*T*_). In Section 2.4.3, we explain in detail how to obtain numerical values for *I*_*T*_ and *V*_*T*_ both from biophysical models and from data. See Fourcaud-Trocmé et al. (2003) for more details about the deterministic version of the model.

Suppose the membrane potential *V* is sampled at *M* time points *t*_0_, *t*_1_, …, *t*_*M*_ with sampling step Δ = *t*_*j*+1_ − *t*_*j*_, such that *v*_*j*_ is the *j*-th sampling point of *V*, over an interval of *L* ms. Hence, the sample is ${\left\{v_{n}\right\}}_{n=0}^{M}$ and the aim is to estimate the time course of *g*_*E*_(*t*) and *g*_*I*_(*t*) from this sample. Below, we will consider subsamples of the data over time windows of length *l* ms, where *l* ≪ *L*. Let *m* = [*l*/Δ] and *M* = [*L*/Δ]. For simplicity and without loss of generality, we assume that *l* and *L* are multiples of Δ, and *m* and *M* are even integers.

Rewrite Equation (1) as:

(2) $$dV=\left(aV^{2}+bV+c\right)dt+\sigma dW_{t},$$

where *W*_*t*_ is a Wiener process, σ scales the noise, and

(3) $$\begin{array}{l} \;a=\frac{\alpha }{C},\\ b(t)=\frac{1}{C}\left(-2\alpha V_{T}-g_{E}(t)-g_{I}(t)\right),\\ c(t)=\frac{1}{C}\left(\alpha V_{T}^{2}-I_{T}+g_{E}(t)V_{E}+g_{I}(t)V_{I}+I_{app}\right). \end{array}$$

Notice that coefficients *b* and *c* are time dependent, whereas coefficient *a* is not. To estimate the constant *a* and the time course of *b* and *c* using only one voltage trace, we first use a sliding window of *l* ms to find an approximate maximum likelihood estimator (MLE) of *a, b* and *c* assuming these constant within each time window. Then, we use the average of the estimators of *a* from all time windows as the final estimate. This estimate of *a* is then used to estimate the time course of *b* and *c*, as explained below. The likelihood function is the product of the transition densities, but these are not known for this model. We therefore approximate the likelihood function using an Euler discretization, and then find the MLE of this approximate model.

We first discretize the diffusion process in Equation (2) as:

(4) $$V_{n+1}\approx V_{n}+(aV_{n}^{2}+b_{n}V_{n}+c_{n})\Delta +\sigma \sqrt{\Delta }\xi_{n+1},$$

where

(5) $$\begin{array}{l} b_{n}=\frac{1}{C}\left(-2\alpha V_{T}-g_{E,n}-g_{I,n}\right),\\ c_{n}=\frac{1}{C}\left(\alpha V_{T}^{2}+g_{E,n}V_{E}+g_{I,n}V_{I}-I_{T}+I_{app}\right), \end{array}$$

with *V*_*n*_ = *V*(*nΔ*), *g*_*E,n*_ = *g*_*E*_(*nΔ*), *g*_*I,n*_ = *g*_*I*_(*nΔ*), and ξ_*n*+1_ follows a Gaussian distribution with mean 0 and variance 1, for *n* = 0, …, *M* − 1. The constants *C, g*_*L*_, *V*_*E*_ and *V*_*I*_ are assumed known from prior experiments (Berg and Ditlevsen, 2013), *I*_*app*_ is controlled by the experimenter, and *I*_*T*_ and *V*_*T*_ are determined from the *V* − *I* curve of the neuron, in a different set of experiments (see Section 2.4.3 for more details on how to obtain these parameters).

Assume we can obtain estimates $â,{\hat{b}}_{n},{ĉ}_{n}$, for *n* = 0, …, *M* − 1, where the hat indicates these are estimates and not the true values. This yields the estimation of the desired parameters, $\hat{\alpha }=âC$, and

(6) $$\begin{array}{l} {\hat{g}}_{E,n}=\frac{A_{n}V_{I}-B_{n}}{V_{I}-V_{E}},\\ {\hat{g}}_{I,n}=\frac{B_{n}-A_{n}V_{E}}{V_{I}-V_{E}}, \end{array}$$

where $A_{n}=-{\hat{b}}_{n}C-2âCV_{T}$ and $B_{n}={ĉ}_{n}C-âCV_{T}^{2}+I_{T}-I_{app}$, for *n* = 0, …, *M* − 1.

The discretized process (4) is Gaussian with conditional mean $V_{n}+\left(aV_{n}^{2}+b_{n}V_{n}+c_{n}\right)\Delta$ and variance σ^2^Δ (conditional on *V*_*n*_). Assume the conductances are approximately stationary for a time window of *l* = *mΔ* ms, i.e., their means are approximately constant, which implies that also the parameters *b* and *c* are approximately constant in this window. Within this sample window, we compute the MLE of parameter θ from the discretized model (see Appendix B for more details). We distinguish two situations: (i) when θ = (*a, b, c*)^*T*^, i.e., both α and the conductances are unknown, in which case the MLE is given by Equation (10); and (ii) when θ = (*b, c*)^*T*^, i.e., only the conductances are unknown, in which case the MLE is given by Equation (11). By moving the sample window, we obtain a discretized sequence for $\hat{\theta }\left(t\right)$, providing a discretized time course of $\hat{\alpha }\left(t\right)$, ĝ_*E*_(*t*) and ĝ_*I*_(*t*) through system (6). The estimation procedure is given in Algorithm 1.

**Algorithm 1:** QIF method. The proposed estimation procedure

1.	Set *n* = *m*/2. While *n* ≤ *M* − *m*/2: use the subsequence ${\left\{v_{j}\right\}}_{j=n-m/2}^{n+m/2}$ to estimate ${\hat{\theta }}_{n}={\left({â}_{n},{\hat{b}}_{n},{ĉ}_{n}\right)}^{T}$ from Equation (10). put ${\hat{\alpha }}_{n}={â}_{n}C$. set *n* = *n*+1.
2.	Put $\hat{\alpha }=\frac{1}{M-m}\overset{M-m/2}{\underset{n=m/2}{\sum }}{\hat{\alpha }}_{n}$.
3.	Set *n* = *m*/2. While *n* ≤ *M* − *m*/2: use the subsequence ${\left\{v_{j}\right\}}_{j=n-m/2}^{n+m/2}$ to estimate ${\hat{\theta }}_{n}={\left({\hat{b}}_{n},{ĉ}_{n}\right)}^{T}$ from Equation (11). use Equation (6) to find ĝ_*E, n*_ and ĝ_*I, n*_. set *n* = *n*+1.
4.	If smoother results are desired, then apply the median filter given in Equation (9) below (or any other filter) to the estimated conductance traces ĝ_*E,n*_ and ĝ_*I,n*_.

Note that the conductances during the first and the last *l*/2 ms are not estimated since otherwise the sliding window would not have sufficient width.

### 2.2. OU method

For the sake of completeness, in this subsection we briefly explain the OU method proposed in Berg and Ditlevsen (2013).

The OU method is based on a stochastic version of the Leaky Integrate-and-Fire model, modeling the subthreshold activity by an Ornstein-Uhlenbeck process. As above, this estimation procedure approximates the membrane potential to be stationary in a given time window. Then, using the MLE within each window (the exact MLE, no discretization is needed in this case), the excitatory and inhibitory conductances are inferred (See Berg and Ditlevsen, 2013 for more details).

The estimation of conductances by using the OU method has been done using the code from Berg (2013) and using the sample window that provides the best estimation results (100 ms for *in silico* data and 300 ms for *in vivo* data).

### 2.3. Oversampling method

For the sake of completeness, in this subsection we briefly explain the oversampling method proposed in Bédard et al. (2012).

The *oversampling method* assumes that the dynamics of the membrane potential has no ionic currents and no noise. It is based on the model $\dot{V}=g_{\alpha }\left(t\right)V+g_{\beta }\left(t\right)$, where *g*_α_ and *g*_β_ are called preconductances which depend linearly on the synaptic conductances. This differential equation is discretized such that the preconductances are represented with half the sampling frequency as that of *V*. Parameters *g*_α_ and *g*_β_ are estimated and used to determine both excitatory and inhibitory conductances. Two thresholds, denoted κ_α_ and κ_β_, need to be defined to avoid possible singularities (See Bédard et al., 2012 for more details).

The estimation of conductances by using the oversampling method has been carried out using the code from Bédard et al. (2012). For the singularity points, the authors recommend that the two thresholds, κ_α_ and κ_β_, should be close to 0.1 when experimental data are analyzed (Bédard et al., 2012). For the *in silico* data, we estimated the conductances using various threshold values, and chose those values providing the best estimation results, also being κ_α_ = κ_β_ ≈ 0.1.

### 2.4. Neuron models

To generate membrane potential traces we use two different neuron models that contain nonlinear subthreshold activity: (a) the QIF model, (Latham et al., 2000; Hansel and Mato, 2001; Gerstner and Kistler, 2002; Fourcaud-Trocmé et al., 2003), and (b) a stellate neuron model (Rotstein et al., 2006). Note that the first model coincides with the model used in the estimation procedure, while the second model is employed to illustrate the robustness of the second order approximation to the nonlinearities. The stellate neuron model is endowed with a spiking mechanism generated by sodium and potassium, and two subthreshold currents, the persistent sodium (NaP) current and the h-current. In the two models, the membrane potential is given by the equation:

(7) $$C\frac{dV(t)}{dt}=f(t,V(t))-I_{syn}(t)+I_{app}+\eta (t),$$

where *f*(*t, V*(*t*)) is a model specific function depending on the leakage current, *I*_*L*_(*t*), and possibly other ionic currents, *V*(*t*) denotes the membrane potential, *C* is the membrane capacitance, *I*_*syn*_(*t*) is the synaptic current, *I*_*app*_ is the applied current, assumed constant for simplicity, and η(*t*) is a white noise process, with zero mean and standard deviation σ, modeling the random arrivals of synaptic input.

The leakage current is modeled as *I*_*L*_(*t*) = *g*_*L*_(*V*(*t*) − *V*_*L*_). The synaptic current is split into the excitatory (*I*_*E*_(*t*)) and the inhibitory (*I*_*I*_(*t*)) currents, which we model as *I*_*E*_(*t*) = *g*_*E*_(*t*)(*V*(*t*) − *V*_*E*_) and *I*_*I*_(*t*) = *g*_*I*_(*t*)(*V*(*t*) − *V*_*I*_).

Common biophysical parameters in both models are set to *C* = 1 μ*F*/cm^2^, $g_{L}=0.1\text{mS}/{\text{cm}}^{2}$, *V*_*L*_ = −65mV, *V*_*E*_ = 0mV, *V*_*I*_ = −80mV and $\sigma =1/C\text{ mV}/\sqrt{\text{ms}}$.

Next, we describe the main features of the two neuron models.

#### 2.4.1. Stochastic version of the quadratic integrate-and-fire model

As a base model for the estimation procedure described in Section 2.1, and in order to generate membrane potential traces using prescribed conductances, we consider the QIF model given by a single equation for the membrane potential in the form (7) with $f\left(t,V\left(t\right)\right)=\alpha {\left(V\left(t\right)-V_{T}\right)}^{2}-I_{T}$, see also Equation (1).

To generate voltage traces from the model, we set the parameters $I_{T}=-1.359\;\mu A/{\text{cm}}^{2}$, *V*_*T*_ = −74.27 mV, $I_{app}\;=-8.7\mu A/{\text{cm}}^{2}$ and α = 0.0067 mS/cm^2^mV.

An example of a trajectory of this model, which we have used to estimate conductances, can be found in **Figure 2C** of Section 3.1.1.

#### 2.4.2. Stellate neuron model

The last model is the medial entorhinal cortex stellate cell model taken from Rotstein et al. (2006). This model has four different currents: the sodium and the potassium currents, which build up the spiking mechanism, and the persistent sodium (NaP) current and the h-current, whose interaction induces subthreshold oscillations independently of the spiking mechanism. The membrane potential is given by Equation (7), with *f*(*t, V*(*t*)) = −*I*_*L*_(*t*) − *I*_*ion*_(*t*) and *I*_*ion*_(*t*) = *I*_*Na*_(*t*) + *I*_*K*_(*t*) + *I*_*NaP*_(*t*) + *I*_*h*_(*t*). We note that the h-current has one fast and one slow component. See Appendix A for more details. The neuron parameters are set to *V*_*T*_ = −58.7379 mV, $I_{T}=-9.496\;\mu A/{\text{cm}}^{2}$, and $I_{app}=-16.9\;\mu A/{\text{cm}}^{2}$. This last value is chosen to be close to the largest current that with high probability do not cause spikes in the model for the given level of noise.

An example of a trajectory of this model, which we have used to estimate conductances, can be found in **Figure 2C** of Section 3.1.2.

#### 2.4.3. Choice of parameters

In the estimation procedure, we need to fix two parameters: the largest input current such that the neuron does not spike, *I*_*T*_, and the corresponding membrane potential, *V*_*T*_. These values are extracted through the *V* − *I* curve, and can be assumed to be known.

To determine the values of these parameters for the *in silico* data generated from the stellate neuron model, we use the V-I bifurcation diagram of the model obtained in the absence of noise and synaptic currents. For this model, there is a threshold value such that for lower values of the input current, the model does not spike, whereas for higher values it does. Parameter *I*_*T*_ corresponds to this threshold value separating nonspiking and spiking regime. Parameter *V*_*T*_ corresponds to the resting value of the membrane potential when the injected current is set to *I*_*T*_. We find $I_{T}=-1.46\;\mu A/{\text{cm}}^{2}$ and *V*_*T*_ = −73.499 mV.

For the *in vivo* data *I*_*T*_ is chosen as the minimum input current that induces spikes by applying different injected currents to the neuron without synaptic inputs. Parameter *V*_*T*_ is the corresponding value in the *V* − *I* curve obtained for the specific neuron (see Section 2.5 to see how to extract the *V* − *I* curve experimentally).

#### 2.4.4. Synaptic drive

We generate traces of conductances following Ornstein-Uhlenbeck processes with sinusoidal drifts, given as solutions to the stochastic differential equation:

(8) $$dx_{t}=\frac{1}{\tau_{x}}\left(x_{0}+\mu_{x}\cos(w_{x}t)-x_{t}\right)dt+\sigma_{x}dW_{t},$$

where *x* denotes either the excitatory (*g*_*E*_) or the inhibitory (*g*_*I*_) conductance. In the simulations, we set τ_*g*_*E*__ = 10ms, τ_*g*_*I*__ = 5ms, $g_{E_{0}}=0.1\text{mS}/{\text{cm}}^{2}$, $g_{I_{0}}=0.14\text{mS}/{\text{cm}}^{2}$, $\mu_{g_{E}}=0.0321\text{mS}/{\text{cm}}^{2}$, $\mu_{g_{I}}=0.0867\text{mS}/{\text{cm}}^{2}$, $w_{g_{E}}=w_{g_{I}}=2\pi /1,000\;{\text{ms}}^{-1}$, $\sigma_{g_{E}}\;=\;0.00064\text{ mS}/\left(\text{c}{\text{m}}^{2}\sqrt{\text{ms}}\right)$, and $\sigma_{g_{I}}\;=\;0.00065\text{mS}/\left({\text{cm}}^{2}\sqrt{\text{ms}}\right)$, unless otherwise stated. These values have been chosen such that amplitudes and frequencies are similar to the estimated traces in the experimental data from Berg and Ditlevsen (2013). The synaptic traces used to generate data from the stellate cell model, have been tripled in order to induce higher subthreshold activity.

The simulated traces of excitatory and inhibitory conductances used for the QIF model can be found in **Figures 2A,B**, while those used for the stellate model can be found in **Figures 3A,B**.

#### 2.4.5. Numerical methods

The stochastic differential equations, both for the synaptic drive and the neuron models, have been solved using the Euler-Maruyama method with a step size of Δ = 0.01ms. Then, data were subsampled every 5th observation to reduce discretization errors. We then get samples with time step 0.05 ms, which is similar to that of the experimental data. We smooth the obtained conductance traces with bandwidth of length *l*_*f*_. We choose the median filter for the smoothing step: for each point *p*: = (*t, x*_*n*_) of a signal *x*, we compute:

(9) $${\tilde{x}}_{n}=\underset{n-m_{f}/2\;\leq \;j\;\leq \;n\;+\;m_{f}/2}{\text{median}}\{x_{j}\},$$

where *m*_*f*_ = [*l*_*f*_/Δ]. We chose this filter since it is often used to clip spikes, but any filter can be used. Unless otherwise stated we set *l*_*f*_ = 50 ms in the simulations.

### 2.5. Experimental data

The experimental data are taken from a previous study, where traces of 25 s of the membrane potential of a motoneuron were measured during different current injections under the same mechanical stimulation (Petersen et al., 2014; Vestergaard and Berg, 2015). The trace analyzed in this paper is shown in **Figure 5B** of Section 3.2. Briefly, the experiments were performed in an integrated spinal cord-carapace preparation from an adult red-eared turtle (*Trachemys scripta elegans*). In the integrated preparation, the spinal cord remains in the spinal canal with the tactile sensory nerves from the carapace intact. The motor nerves are carefully transected to avoid muscle movements and dissected out for electroneurogram recordings. A scratch reflex was activated by mechanical somato-sensory stimulation of selected regions on the carapace, which induced motor network activity of ~20 s duration. Intracellular recordings in current-clamp mode were obtained from a motoneuron in segment D10. Data were sampled at 20 kHz, i.e., the time step between observations is Δ = 0.05 ms.

The determination of *I*_*T*_ and *V*_*T*_ is not exact, and these values might induce a bias in the estimated conductances, and in particular, could give rise to negative conductance estimates. Since conductance cannot be negative, the sign of *g*_*I*_ and/or *g*_*E*_ provides a means to verify the initial estimate of *I*_*T*_ and *V*_*T*_.

**Figure 1:**
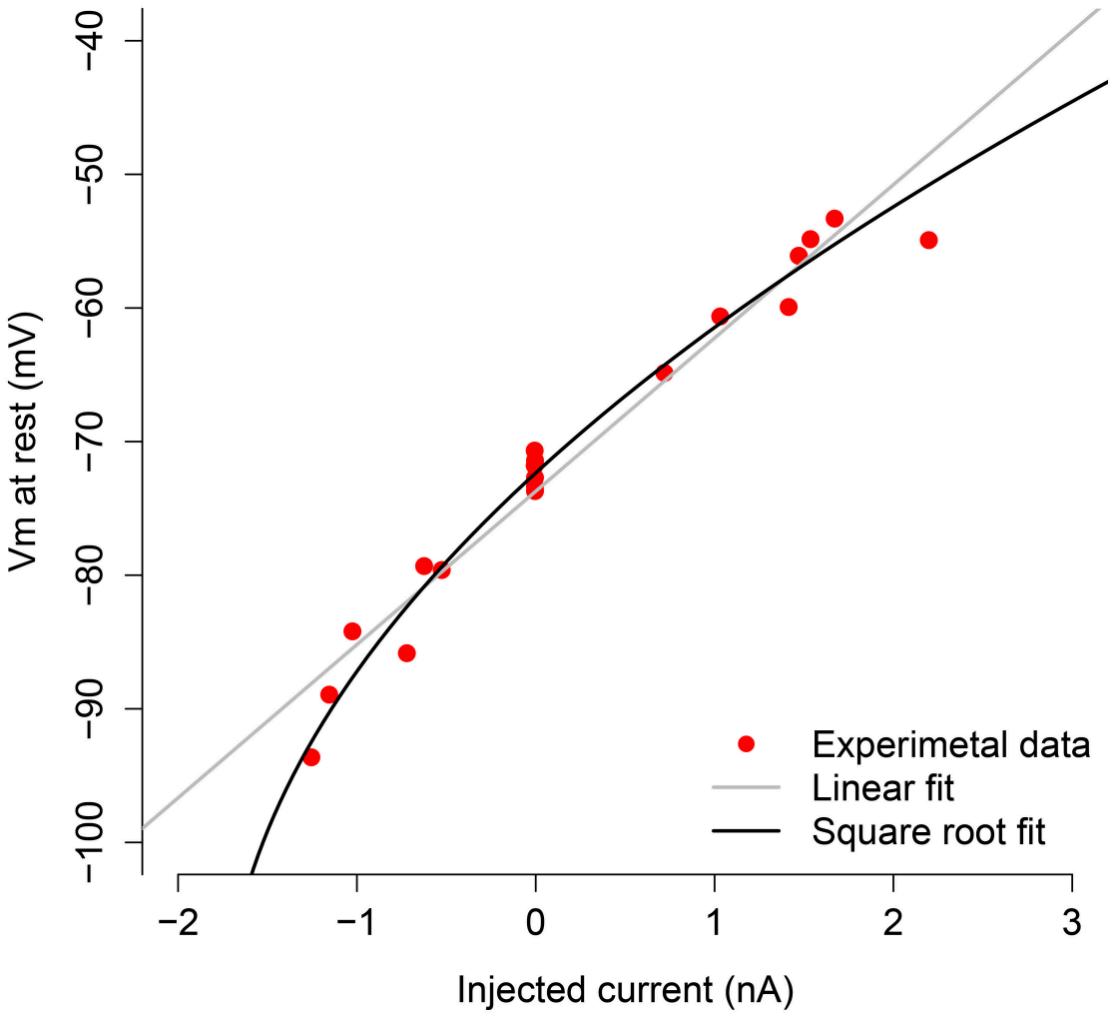
V-I curve of the measured motoneuron. The red dots are the subthreshold *V* − *I* relations obtained by applying different current levels to the motoneuron. The black line shows the square root fit to these points, and the gray line depicts the linear fit.

In Figure 1, we depict the *V* − *I* relationship (red dots) obtained by injecting different levels of current into the neuron in absence of synaptic activity. We made a linear fit (gray line) and a square root fit (black line) of the *V* − *I* points. The square root fit is clearly chosen above the linear fit using either AIC or BIC criteria, since differences are both larger than 7 (ΔAIC = 9.2, ΔBIC = 8.1). This value is suggested in Burnham and Anderson (2002) as the critical value for the less plausible model to have considerably less support in the data compared with the better model.

The square root approximation (squared approximation as a function of *V*) is given by $I_{app}\left(V\right)=0.00095V^{2}+0.22V+11$. Since the last input current for which the neuron did not spike was −0.515 μ*A*/cm^2^, we set $I_{T}=-0.515\;\mu A/{\text{cm}}^{2}$, which corresponds to *V*_*T*_ = −79.926 mV from the *V* − *I* curve. The remaining neuron parameters have been set to *V*_*L*_ = −77 mV, *V*_*I*_ = −79 mV, *V*_*E*_ = 0 mV, $g_{L}=0.026\;{\text{mS}/\text{cm}}^{2}$, *I*_*app*_ = -1.24 *nA*, and *C* = 1 μ*F*/cm^2^; these values have been obtained from the actual data by using the code in Berg (2013).

## 3. Results

In this section, we present the estimation results from simulated (*in silico*) and experimental (*in vivo*) data by using the estimation procedure given in Algorithm 1. The simulated data consist of membrane potential traces generated from each of the two neuron models: the QIF model and the stellate neuron model, described in Section 2. Note that the estimation procedure assumes that the data are generated from the QIF model, and thus, results from the other model show how robust the estimation procedure is to model misspecification. The experimental data are obtained from a motoneuron as described in Section 2.5. Finally, the results obtained from the stellate neuron model as well as from experimental recordings, are compared to both the oversampling method and the OU method.

### 3.1. Estimation results from simulated data

#### 3.1.1. Results for the QIF model

**Figure 2:**
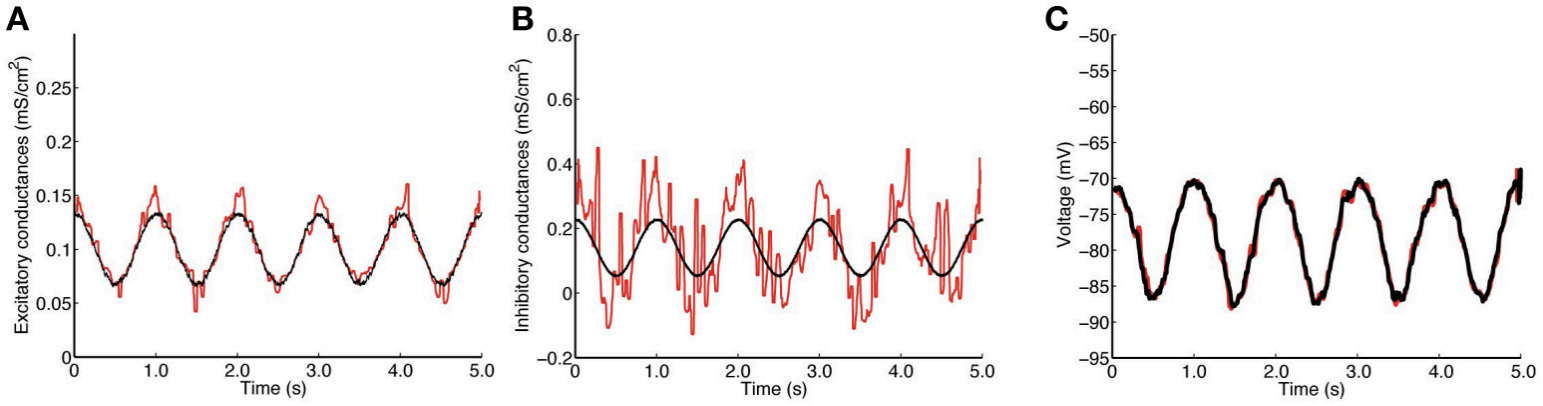
Estimation by the QIF method of the conductances from data generated from the QIF neuron model. **(A)** True (black) and estimated (red) excitatory conductances. **(B)** true (black) and estimated (red) inhibitory conductances. **(C)** simulated voltage using the true conductances (black) and the estimated conductances (red). The data have been obtained each *dt* = 0.05 ms and the MLE is applied with a *l* = 50 ms sliding window. The estimated conductances have been filtered by the median filter using a sample window of *l*_*f*_ = 50 ms. The neuron parameters are given in Section 2.4.1, and the synaptic drive description is given in Section 2.4.4. Estimation has been performed using the QIF method in Algorithm 1. See Figure S1 in Supplementary Material for a plot of true vs. estimated conductances.

Figure 2 shows the estimation results when the membrane potential is generated from the QIF model, setting α = 0.0067. This parameter was estimated to $\hat{\alpha }=0.0077$. Panel A depicts the true (black curve) and the estimated (red curve) excitatory conductances, and panel B shows the true (black) and the estimated (red) inhibitory conductances. The true excitatory trace is well approximated by the estimated trace, whereas the estimation of the inhibitory conductances only captures the overall shape and level, and is much more noisy. This is because the membrane potential is close to the inhibitory reversal potential, whereas it is far from the excitatory reversal potential, and thus, the synaptic drive is higher for excitation. This was also shown in Berg and Ditlevsen (2013), where analytic expressions for approximations of the variance of the estimators were derived from the Fisher Information matrix. Panel C shows the true membrane potential (black curve) and the reconstructed voltage by using the estimated conductances (red curve). The actual voltage is well reproduced by the reconstructed one.

#### 3.1.2. Results for the stellate neuron model

**Figure 3:**
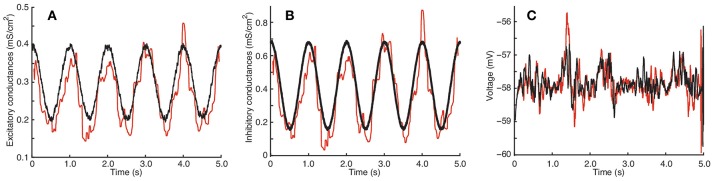
Estimation by the QIF method of conductances from data generated from the stellate cell model. **(A)** True (black) and estimated (red) excitatory conductances. **(B)** True (black) and estimated (red) inhibitory conductances. **(C)** Simulated voltage using the true conductances (black) and the estimated conductances (red). The data were generated each *dt* = 0.05 ms and the MLE sample window was *l* = 100 ms. The estimated conductances have been filtered by the median filter using a sample window of *l*_*f*_ = 50 ms. The neuron parameters are given in Section 2.4.2, and the synaptic drive description is given in Section 2.4.4; additionally, for this model, we have tripled the conductance traces obtained from (8) in order to induce higher subthreshold activity. Estimation has been performed using the QIF method given in Algorithm 1. See Figure S3 in Supplementary Material for a plot of when the sliding window is *l* = 50 ms.

Results for the stellate neuron model are depicted in Figure 3. Panels are as in Figure 2.

The excitatory and inhibitory conductances are well approximated; however, as in the QIF model, the estimate of the excitatory conductance trace is more accurate. It can be appreciated how well the reconstructed voltages match the true voltages. The estimated value of α is 0.0100.

Surprisingly, the estimated conductances for this model are nearly as good as the original QIF model, and show robustness of the method. It also indicates that a quadratic approximation seems to be sufficient to capture the nonlinearities caused by the ionic currents in this more biophysically realistic model.

Note that, for this model, the specified conductances do not induce large fluctuations in the membrane potential, but even these small effects provide sufficient information to recover the overall structure of the conductances. If the excitatory conductance is increased relative to the inhibitory conductance, larger oscillations in the membrane potential are seen, comparable to the QIF model (results not shown). It is remarkable that even so small fluctuations in the membrane potential are sufficient to trace and separate the excitatory and inhibitory dynamics.

#### 3.1.3. Effects of the variability of the quadratic term, sliding window size and injected current

**Figure 4:**
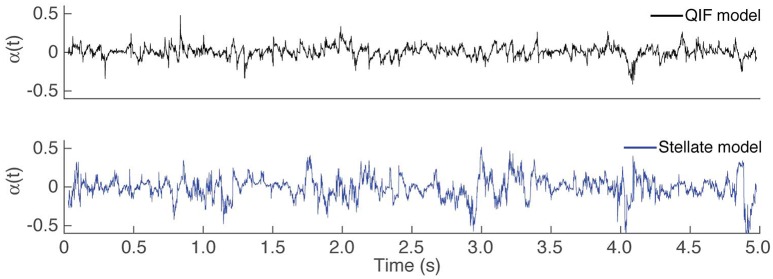
Time course of $\hat{\alpha }\left(t\right)$ using Algorithm 1. Estimated time varying α in the first step of Algorithm 1 for the different *in silico* models: QIF model in the top panel (black trace) and the stellate model in the bottom panel (blue trace).

During the estimation procedure described in Section 2.1, first a time varying estimate of α is obtained, then the average is used as the final estimate of the constant value of α. It might be of interest to study how α as either a time-varying or a constant parameter affects the estimates of the conductances. Figure 4 depicts the estimated time varying α over the observation interval for both computational neuron models. The variability is relatively small for the QIF model, and slightly larger for the stellate neuron model, probably because the membrane potential in this case is less oscillating, thus containing less information about the dynamics.

The error of a given estimate is assessed by the mean squared error (MSE). The MSE of estimating **x** by **y** is defined as (**x** − **y**)^2^/*M*, where **x** and **y** are either excitatory or inhibitory conductances or the membrane potential, and *M* is the length of **x**.

To assess how the estimates are affected by imposing α constant, the MSEs obtained for the stellate model are given in Table 1 from (i) letting α be time varying; and (ii) forcing α to be constant. The errors of the estimated conductances are smaller when α is constant, in agreement with the QIF method. Moreover, imposing the second order term to be constant probably makes the estimates of the time varying conductances more robust and less noisy.

**Table 1:** Mean squared errors of the excitatory and inhibitory conductances with α constant or time varying.

**Algorithm**	***g*_*E*_**	***g*_*I*_**
QIF method with α constant	2.03·10^−3^	9.44·10^−3^
QIF method with α time varying	2.38·10^−3^	1.27·10^−2^

*Data have been generated from the stellate neuron model described in Section 2.4.2 with synaptic drive given in Section 2.4.4. The window size used in both cases is l = 100 ms. The estimated conductances have been filtered by the median filter using a sample window of l_f_ = 50 ms. The MSEs are given for excitatory and inhibitory conductances letting α be time varying or constant*.

During the estimation procedure described in Section 2.1 (Algorithm 1), some values need to be chosen: (i) the size of the sliding window, within which conductances are supposed to be stationary; and (ii) the applied current that is injected to prevent the cell from spiking.

When choosing the size of the sample window, there is a trade-off between the assumption about stationarity and accuracy: the window has to be small for the conductances to be approximately stationary but large enough for the MLE to produce low errors. In Table 2, errors of the conductances and the reconstructed membrane potential are quantified by MSE for different window sizes. Errors are computed for the QIF method applied to simulated data from the stellate cell model described in Section 2.4.2 with synaptic drive given in Section 2.4.4. The window size with smallest error is around 100 ms.

**Table 2:** Mean squared errors of the QIF method for different window sizes.

**Sample window (ms)**	***g*_*E*_**	***g*_*I*_**	***v***
*l* = 10	2.12·10^−2^	1.59·10^−1^	2.54·10^−1^
*l* = 25	4.01·10^−3^	2.96·10^−2^	2.14·10^−1^
*l* = 50	2.40·10^−3^	1.46·10^−2^	1.15·10^−1^
*l* = 75	1.92·10^−3^	9.59·10^−3^	1.08·10^−1^
*l* = 100	2.03·10^−3^	9.44·10^−3^	7.34·10^−2^
*l* = 150	2.14·10^−3^	9.46·10^−3^	6.71·10^−2^
*l* = 200	2.19·10^−3^	9.54·10^−3^	9.54·10^−2^

*Data have been generated from the stellate cell model described in Section 2.4.2 with synaptic drive given in Section 2.4.4. The estimated conductances have been filtered by the median filter using the same window size as the estimation procedure. The MSEs are given for excitatory conductances (first column), inhibitory conductances (second column), and the membrane potential (third column). Similar effects are observed for the OU method, but with higher MSE, see Table 3*.

Finally, the estimation errors are also sensitive to the amount of applied current *I*_*app*_. Lower values of *I*_*app*_ hyperpolarize the membrane potential away from the threshold, thus separating the neuron dynamics from the regime where the QIF method is more advantageous. In the simulations, the maximal applied current for which the neuron does not exhibit spikes in the presence of noise and synaptic currents was injected. To study the effect of lowering the applied current, the MSE for different values of *I*_*app*_ injected into the stellate cell model is provided in Table 3. Moving the dynamics away from the threshold induces a loss of accuracy. However, a large negative current needs to be applied in order to see a large increase in MSE, and, even in those cases, the QIF method still performs better than the OU method. We repeated the simulations with different random seeds and results were qualitatively the same (see Supplementary Material, Table S2).

**Table 3:** Mean squared errors of the QIF and OU methods applied to the stellate cell model for different applied currents.

**$I_{app}\;\left(\mu A/{\text{cm}}^{2}\right)$**	**QIF method**		**OU method**	
	***g*_*E*_**	***g*_*I*_**	***g*_*E*_**	***g*_*I*_**
−16.9	2.03·10^−3^	9.44·10^−3^	1.13·10^−2^	1.15·10^−1^
−17.9	1.78·10^−3^	1.24·10^−2^	1.29·10^−2^	1.28·10^−1^
−19.9	1.23·10^−3^	1.01·10^−2^	1.91·10^−2^	1.01·10^−1^
−28.9	7.99·10^−3^	2.21·10^−2^	5.21·10^−2^	1.23·10^−1^

*Data have been generated from the stellate cell model described in Section 2.4.2 with synaptic drive given in Section 2.4.4. The window size used in both methods is l = 100 ms. The estimated conductances have been filtered by the median filter using a sample window of l_f_ = 50 ms. The MSEs are given for excitatory conductances, g_E_, and inhibitory conductances, g_I_*.

To evaluate the effect of possible measurement noise on the membrane potential, we repeated the analyses on the simulated data from the stellate model by adding independent normally distributed variables with mean 0. The QIF method seems robust in the presence of small levels of measurement noise. For a standard deviation of 0.1 mV, the MSEs increase 2–3 times on the conductances, whereas the MSEs increase slightly less for the OU method, but the QIF still performs better. However, for a large standard deviation of 0.5 mV, the QIF methods breaks down, while the OU method still performs well. Hence, if measurement noise is large compared to the nonlinear effects, the OU method is more robust and will perform better, whereas for a small measurement noise, the QIF method is recommended.

### 3.2. Estimation results from experimental data

**Figure 5:**
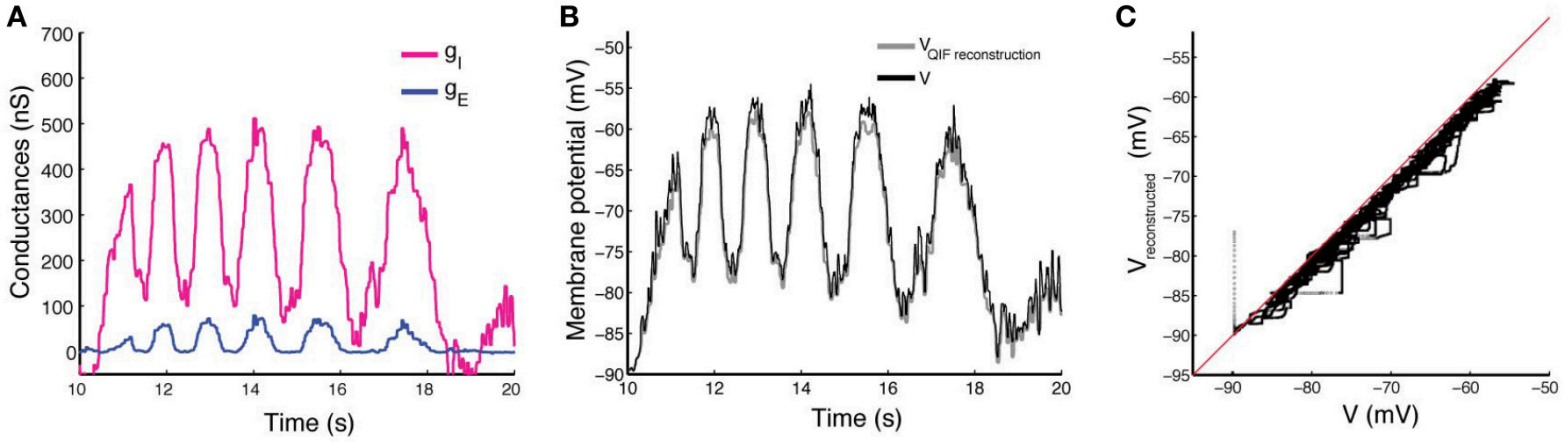
Estimation by the QIF method of conductances from experimental data and reconstruction of the membrane potential. **(A)** Estimated excitatory (blue) and inhibitory (magenta) conductances from the membrane potential obtained from *in vivo* experiment. The QIF method has been applied using a sliding window of *l* = 100 ms. **(B)** The recorded membrane potential (black line) and reconstructed membrane potential obtained by using the estimated values of conductance traces and the estimated α parameter (gray line). **(C)** The reconstructed vs. the true voltage. Estimation has been performed using the QIF method in Algorithm 1.

In Figure 5 we show the results obtained with the estimation procedure on experimental data described in Section 2.5. The magenta line shows the estimated inhibitory conductances whereas the blue line shows the estimated excitatory conductances. Contrary to the *in silico* data, we have no information on the true input conductances, and thus, they can not be compared.

As shown in Figure 5A, both conductance traces follow the shape of the membrane potential, suggesting that the network generates the motor activity by balanced inhibition and excitation. Moreover, the QIF method provided an estimated value of the quadratic coefficient of α ≈ 0.1094. The reconstructed membrane potential dynamics using the QIF model with the estimated conductances as synaptic input is shown in Figure 5B, and shows good agreement with the true voltage trace, though it is slightly underestimated as can be appreciated in the scatter plot of Figure 5C. In this case, the MSE obtained is 3.04. We have also reconstructed the membrane potential using the OU method obtaining a MSE of 9.62.

### 3.3. Comparison with other procedures

In this section we compare the QIF method with other existing procedures that estimate conductances from a single trace, namely the OU method and the oversampling method, described in Sections 2.2 and 2.3. The estimation is conducted using the codes of these procedures published by the authors (Bédard et al., 2012; Berg, 2013). We first tested both codes on the linear neuronal models that the methods assume, using the prescribed conductances given in Section 2.4.4, and found similar results as the original papers.

Comparisons between the three different estimation procedures have been performed on the stellate cell model.

#### 3.3.1. Comparisons between the QIF, OU and oversampling methods for *in silico* data generated from the stellate model.

**Figure 6:**
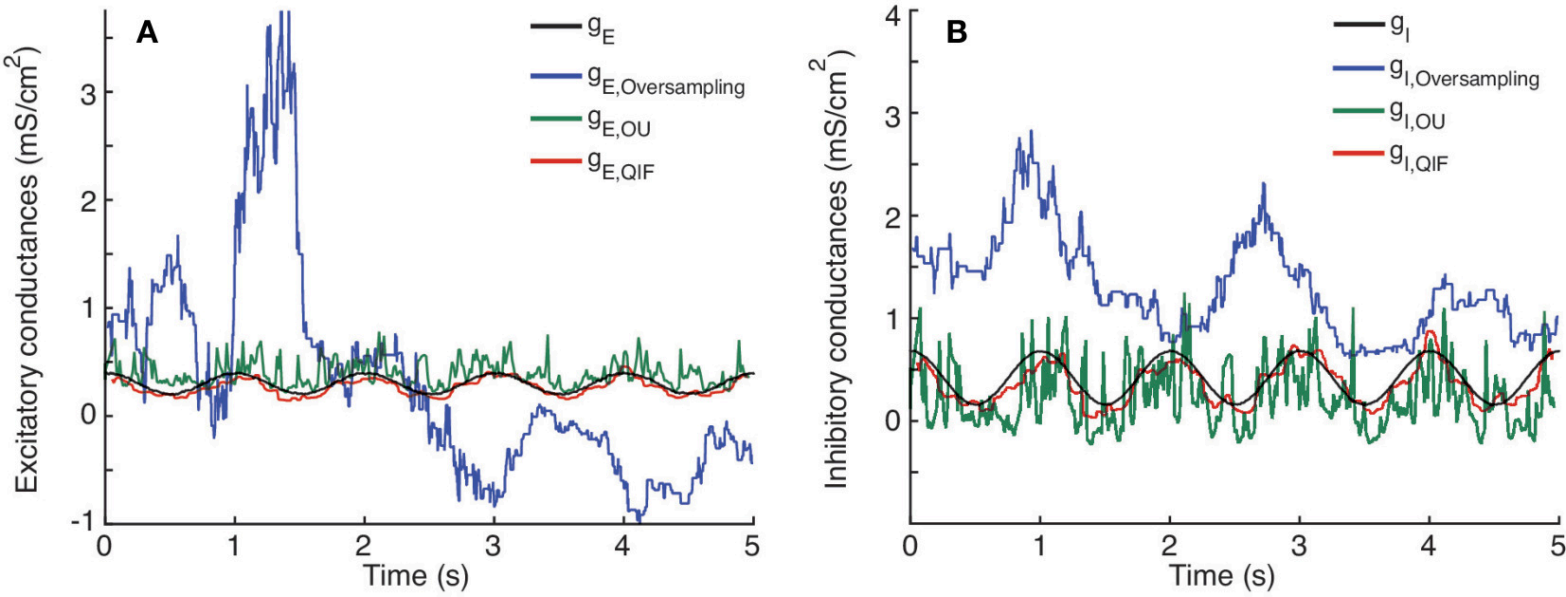
Comparison between single-trial estimation procedures; QIF, OU and oversampling method on data generated from the stellate model. Prescribed and estimated synaptic input generated from Equation (8). **(A)** Excitatory conductance. **(B)** Inhibitory conductance. The sample window used in the QIF and the OU methods is *l* = 100 ms. In the oversampling method, parameter values are κ_α_ = κ_β_ = 0.1. For the QIF method, the estimated conductances have been filtered by the median filter using a sample window of *l*_*f*_ = 50 ms.

In Figure 6 the results obtained with the QIF method are compared to the OU and the oversampling method, using simulated data from the stellate cell model described in Section 2.4.2.

The time courses of the true conductances are depicted together with the estimates from the three methods. Both the QIF and the OU show acceptable fits. The QIF method provides a slight improvement on the estimated excitatory conductance compared to the OU method, whereas the inhibitory conductance is considerable better estimated with the QIF compared to the OU method. The oversampling method fails and should not be used for this type of data.

To quantify and compare the errors of the different estimation procedures, the MSEs and bias are shown in Table 4. The MSE for the QIF method is smaller than those obtained using either the OU method (by one order of magnitude) and the oversampling method (by two orders of magnitude). The MSE is smaller for the QIF method compared to the OU method mainly due to decreased variance, whereas the bias is comparable. For this example, the QIF has lower bias, but for other examples of Hodgkin-Huxley type models the bias is slightly smaller for the OU method (see Supplementary Material). The difference between the QIF and the OU MSEs is consistent throughout the different applied currents (see Table 3).

**Table 4:** Mean squared errors and bias of the different estimation procedures.

**Estimation procedure**	***g*_*E*_ MSE**	***g*_*I*_ MSE**	***g*_*E*_ bias**	***g*_*I*_ bias**
QIF method	2.03·10^−3^	9.44·10^−3^	3.44·10^−2^	5.98·10^−2^
OU method	1.13·10^−2^	1.15·10^−1^	−6.55·10^−2^	2.61·10^−1^
Oversampling method	9.79·10^−1^	1.02	−1.71·10^−2^	−8.85·10^−1^

*Data have been generated from the modified stellate cell model described in Section 2.4.2 with synaptic drive given in Section 2.4.4. The window size used in the QIF and the OU methods is l = 100 ms. The estimated conductances have been filtered by the median filter using a sample window of l_f_ = 50 ms. In the oversampling method, parameter values are κ_α_ = κ_β_ = 0.1. For the QIF method, the MSE are given for excitatory conductances (second column) and inhibitory conductances (third column). Bias is given for excitatory conductances (third column) and inhibitory conductances (fourth column)*.

#### 3.3.2. Comparison of the methods on experimental data

**Figure 7:**
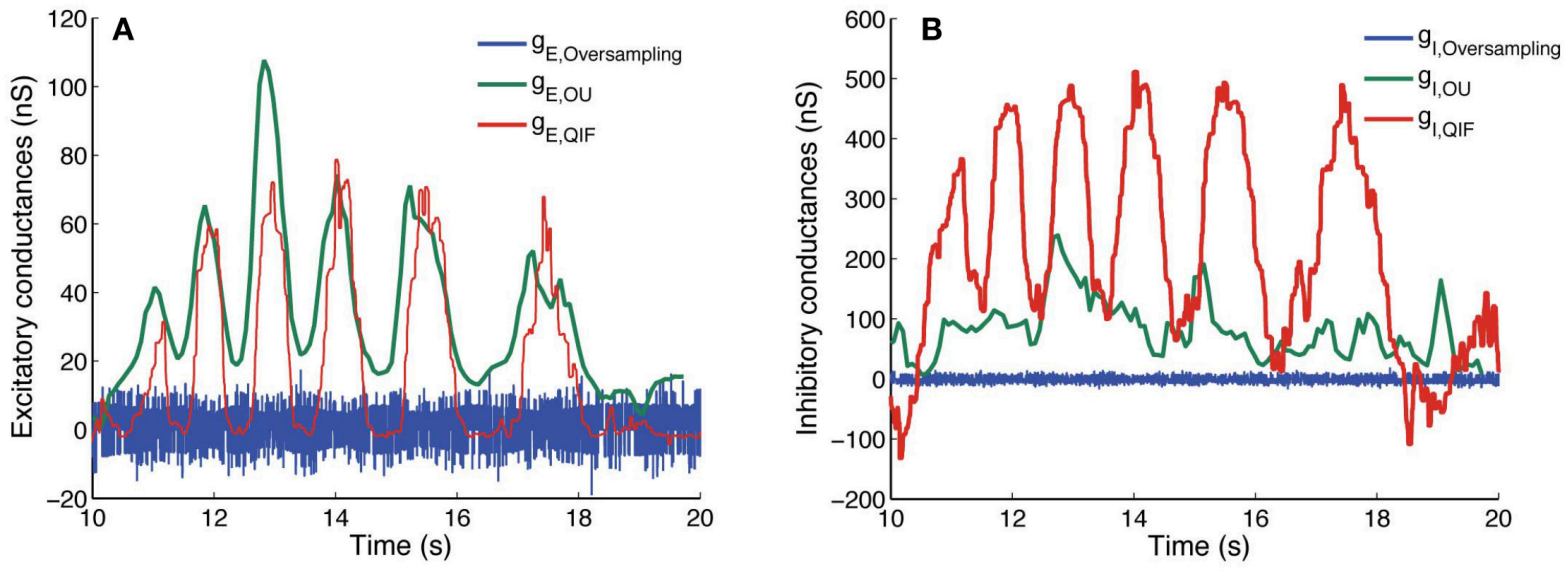
Estimated conductances from experimental data using the QIF, OU, and oversampling methods. Estimated conductances from the *in vivo* data described in Section 2.5, for the QIF method (red curves), the OU method (green curves) and the oversampling method (blue curves). The time window is *l* = 50 ms for the QIF method and *l* = 300 ms for the OU method. In the oversampling method, values are κ_α_ = κ_β_ = 0.1. For the QIF method, the estimated conductances have been filtered by the median filter using a sample window of *l*_*f*_ = 50 ms. **(A)** Excitatory conductances. **(B)** Inhibitory conductances.

Finally, we apply the three methods on the experimental data described in Section 2.5. Results are depicted in Figure 7. The results obtained using the three different methods for the experimental data are very different. Only the excitatory conductances estimated with the OU and the QIF method seem to follow a similar pattern, even though there is a vertical shift between the two. The oversampling method presents a similar pattern as the ones obtained in Bédard et al. (2012, Figure 2), where they present results previous to suppressing singular points. In Figure 7, we show the best results we have obtained varying κ_α_ and κ_β_ even after this suppression. Therefore, it seems that this method is not able to provide plausible estimates from these data.

Both the oversampling and the OU method assume a linear behavior of the subthreshold activity. However, the *V* − *I* curve obtained from the experimental recordings (Figure 1) is better fitted by a square root regression than a linear one, thus revealing the existence of nonlinear subthreshold activity. Thus, we believe that the results obtained with the QIF method are closer to the real conductances than the other methods, which was also the case for the *in silico* data.

## 4. Discussion

In this paper we propose a new method to estimate synaptic conductances from voltage traces in the subthreshold regime, based on a combination of methods proposed in Berg and Ditlevsen (2013) and Vich and Guillamon (2015). We have shown that the method overcomes some of the drawbacks of existing methods pointed out in the Introduction. The method only uses a single-trial voltage trace, thus avoiding the requirement that synaptic conductances are identical across trials. Furthermore, since it is based on a quadratic model with stochastic terms, it is able to account for the nonlinearities in the subthreshold regime while it incorporates noise.

When the membrane potential is far below the threshold, we sometimes obtain negative estimates with the QIF method for the inhibitory conductances (Figures 5A, 7B), which is not as pronounced for the OU method. This suggests to combine linear and quadratic methods when the membrane potential is hyperpolarized. However, in excitability conditions, the method improves both the OU method (Berg and Ditlevsen, 2013), and the oversampling method (Bédard et al., 2012). In particular, we have tested it on intracellular recordings in current-clamp mode of spinal motoneurons, and the results show reasonable estimates of rhythmic activity of both excitatory and inhibitory drive. Interestingly, for this specific case, we do not observe any push-pull arrangement that signals a balanced input, which could still be possible at a population level.

For the *in silico* data, where prescribed conductances have been injected into the cells, both the excitatory and the inhibitory conductances are well estimated. We tested the method on data generated from the QIF model, as well as from the stellate model, which is a more biophysical realistic model and shows the robustness of the method to model misspecifications. In the Supplementary Material we furthermore show computations with a modified pyramidal cell model, which confirms the validity of the results. Moreover, the QIF method exhibits a significant improvement with respect to the other two methods tested in this paper, mostly compared to the oversampling method, thus underpinning the necessity of nonlinear strategies to estimate conductances also in the subthreshold regime, and showing improved results when quadratic terms are considered in the estimation procedure. The significant improvement compared to the oversampling procedure could be explained by the fact that the oversampling method does not consider neither ionic currents nor noise. It is worth mentioning as well that the QIF method has been successfully applied to a realistic neuron model having different subthreshold-activated currents, thus validating the QIF model as a reference model for subthreshold activity in this problem.

For the *in vivo* recordings, we see first that the *V* − *I* curve provided by the data is better fitted by a quadratic function than by a linear one, providing experimental evidence of the suitability of the quadratic approximation. Even though actual conductances are unknown, we have seen that the pattern estimated with the stochastic quadratization is more coherent that those obtained with the other linear procedures, at least if we expect the conductances to follow dynamics similar to the membrane potential. Moreover, the oversampling method has not been able to extract the conductances from these data, again probably a consequence of both the noise and the nonlinearities.

In summary, we propose a novel reliable strategy to infer synaptic activity received by a cell in the subthreshold regime. Apart from the constraint of the activity being in the subthreshold regime, not too far from the threshold, the method is robust enough to be applied to neurons whose V-I curve follows a quadratic shape. The next challenge is to test it on other electrophysiological data from different brain areas and animals, as well as comparing with already existing conductance traces obtained in the literature by means of linear estimation methods. On the other hand, the Exponential Integrate-and Fire (EIF) model and other IF extended models (see for instance; Fourcaud-Trocmé et al., 2003; Brette and Gerstner, 2005) are able to capture a wide repertoire of dynamics exhibited by conductance-based models. However, in this paper we treat only the subthreshold regime, and the QIF model captures the basic dynamics of conductance-based models. Further adaptations to more comprehensive IF models might improve estimates in a broader range of dynamic regimes. These extensions constitute an interesting challenging research direction to pursue.

## Ethics statement

This study was carried out in accordance with the recommendations of the surgical procedures complied with Danish legislation. The protocol was approved by the controlling body under the Ministry of Justice.

## Author contributions

All authors conceptualized the research. RB designed and performed experiments. CV performed the analysis and prepared figures. All authors interpreted the results. CV, SD, and AG wrote the paper. All authors approved the final version of the paper.

### Conflict of interest statement

The authors declare that the research was conducted in the absence of any commercial or financial relationships that could be construed as a potential conflict of interest.

## References

[B1] Anderson J. S. Carandini M. Ferster D. (2000). Orientation tuning of input conductance, excitation, and inhibition in cat primary visual cortex. J. Neurophys. 84, 909–926. 10938316 10.1152/jn.2000.84.2.909

[B2] Bédard C. Béhuret S. Deleuze C. Bal T. Destexhe A. (2012). Oversampling method to extract excitatory and inhibitory conductances from single-trial membrane potential recordings. J. Neurosci. Methods 210, 3–14. 10.1016/j.jneumeth.2011.09.010 21968037

[B3] Berg R. W. (2013). Available online at: www.mathworks.com/matlabcentral/fileexchange/41774-synapticconductance

[B4] Berg R. W. Alaburda A. Hounsgaard J. (2007). Balanced inhibition and excitation drive spike activity in spinal half-centers. Science 315, 390–393. 10.1126/science.1134960 17234950

[B5] Berg R. W. Ditlevsen S. (2013). Synaptic inhibition and excitation estimated via the time constant of membrane potential fluctuations. J. Neurophysiol. 110, 1021–1034. 10.1152/jn.00006.2013 23636725

[B6] Borg-Graham L. Monier C. Frégnac Y. (1998). Visual input evokes transient and strong shunting inhibition in visual cortical neurons. Nature 393, 369–373. 10.1038/30735 9620800

[B7] Brette R. Gerstner W. (2005). Adaptive exponential integrate-and-fire model as an effective description of neuronal activity. J. Neurophysiol. 94, 3637–3642. 10.1152/jn.00686.2005 16014787

[B8] Burnham K. P. Anderson D. R. (2002). Model Selection and Multimodel Inference: A Practical Information-Theoretic Approach. New York, NY: Springer.

[B9] Closas P. (2014). Sequential Estimation of Neural Models by Bayesian Filtering. Diploma thesis, FME-Univ. Politènica de Catalunya.

[B10] Destexhe A. Babloyantz A. (1993). A model of the inward current *i*_*h*_ and its possible role in thalamocortical oscillations. Neuroreport 4, 223–226. 10.1097/00001756-199302000-00028 8453063

[B11] Ditlevsen S. Samson A. (2014). Estimation in the partially observed stochastic morris-lecar neuronal model with particle filter and stochastic approximation methods. Ann. Appl. Stat. 8, 674–702. 10.1214/14-AOAS729

[B12] Ermentrout G. B. Kopell N. (1986). Parabolic bursting in an excitable system coupled with a slow oscillation. SIAM J. Appl. Math. 46, 233–253. 10.1137/0146017

[B13] Fourcaud-Trocmé N. Hansel D. van Vreeswijk C. Brunel N. (2003). How spike generation mechanisms determine the neuronal response to fluctuating inputs. J. Neurosci. 23, 11628–11640. 14684865 10.1523/JNEUROSCI.23-37-11628.2003 PMC6740955

[B14] Gerstner W. Kistler W. (2002). Spiking Neuron Models. Cambridge: Cambridge University Press.

[B15] Guillamon A. McLaughlin D. W. Rinzel J. (2006). Estimation of synaptic conductances. J. Physiol. 100, 31–42. 10.1016/j.jphysparis.2006.09.010 17084599 PMC2042540

[B16] Hansel D. Mato G. (2001). Existence and stability of persistent states in large neuronal networks. Phys. Rev. Lett. 86:4175. 10.1103/PhysRevLett.86.4175 11328124

[B17] Jahn P. Berg R. W. Hounsgaard J. Ditlevsen S. (2011). Motoneuron membrane potentials follow a time inhomogeneous jump diffusion process. J. Comput. Neurosci. 31, 563–579. 10.1007/s10827-011-0326-z 21479618 PMC3232348

[B18] Jolivet R. Lewis T. J. Gerstner W. (2004). Generalized integrate-and-fire models of neuronal activity approximate spike trains of a detailed model to a high degree of accuracy. J. Neurophysiol. 92, 959–976. 10.1152/jn.00190.2004 15277599

[B19] Kobayashi R. Nishimaru H. Nishijo H. (2016). Estimation of excitatory and inhibitory synaptic conductance variations in motoneurons during locomotor-like rhythmic activity. Neuroscience 335, 72–81. 10.1016/j.neuroscience.2016.08.027 27561702

[B20] Kobayashi R. Tsubo Y. Shinomoto S. (2009). Made-to-order spiking neuron model equipped with a multi-timescale adaptive threshold. Front. Comput. Neurosci. 3:9. 10.3389/neuro.10.009.2009 19668702 PMC2722979

[B21] Lankarany M. Heiss J. E. Lampl I. Toyoizumi T. (2016). Simultaneous bayesian estimation of excitatory and inhibitory synaptic conductances by exploiting multiple recorded trials. Front. Comput. Neurosci. 10:110. 10.3389/fncom.2016.00110 27867353 PMC5095134

[B22] Lankarany M. Zhu W. P. Swamy M. N. S. Toyoizumi T. (2013). Inferring trial-to-trial excitatory and inhibitory synaptic inputs from membrane potential using Gaussian mixture Kalman filtering. Front. Comput. Neurosci. 7:109. 10.3389/fncom.2013.00109 24027523 PMC3759749

[B23] Latham P. E. Richmond B. J. Nelson P. G. Nirenberg S. (2000). Intrinsic dynamics in neuronal networks. I. Theory. J. Neurophysiol. 83, 808–827. 10669496 10.1152/jn.2000.83.2.808

[B24] Monier C. Fournier J. Fregnac Y. (2008). *In vitro* and *in vivo* measures of evoked excitatory and inhibitory conductance dynamics in sensory cortices. J. Neurosci. Methods 169, 323–365. 10.1016/j.jneumeth.2007.11.008 18215425

[B25] Paninski L. Vidne M. DePasquale B. Ferreira D. G. (2012). Inferring synaptic inputs given a noisy voltage trace via sequential monte carlo methods. J. Comput. Neurosci. 33, 1–19. 10.1007/s10827-011-0371-7 22089473

[B26] Petersen P. C. Vestergaard M. Jensen K. H. Berg R. W. (2014). Premotor spinal network with balanced excitation and inhibition during motor patterns has high resilience to structural division. J. Neurosci. 34, 2774–2784. 10.1523/JNEUROSCI.3349-13.2014 24553920 PMC6608521

[B27] Pospischil M. Piwkowska Z. Bal T. Destexhe A. (2009). Extracting synaptic conductances from single membrane potential traces. Neuroscience 158, 545–552. 10.1016/j.neuroscience.2008.10.033 19027831

[B28] Rotstein H. (2015). Subthreshold amplitude and phase resonance in models of quadratic type: Nonlinear effects generated by the interplay of resonant and amplifying currents. J. Comput. Neurosci. 38, 325–354. 10.1007/s10827-014-0544-2 25586875

[B29] Rotstein H. Oppermann T. White J. Kopell N. (2006). The dynamic structure underlying subthreshold oscillatory activity and the onset of spikes in a model of medial entorhinal cortex stellate cells. J. Comput. Neurosci. 21, 271–292. 10.1007/s10827-006-8096-8 16927211

[B30] Rudolph M. Piwkowska Z. Badoual M. Bal T. Destexhe A. (2004). A method to estimate synaptic conductances from membrane potential fluctuations. J. Neurophysiol. 91, 2884–2896. 10.1152/jn.01223.2003 15136605

[B31] Vestergaard M. Berg R. W. (2015). Divisive gain modulation of motoneurons by inhibition optimizes muscular control. J. Neurosci. 35, 3711–3723. 10.1523/JNEUROSCI.3899-14.2015 25716868 PMC6605555

[B32] Vich C. Guillamon A. (2015). Dissecting estimation of conductances in subthreshold regimes. J. Comput. Neurosci. 39, 271–287. 10.1007/s10827-015-0576-2 26432075

[B33] Wehr M. Zador A. M. (2003). Balanced inhibition underlies tuning and sharpens spike timing in auditory cortex. Nature 426, 442–446. 10.1038/nature02116 14647382

[B34] Yasar T. B. Wright N. C. Wessel R. (2016). Inferring presynaptic population spiking from single-trial membrane potential recordings. J. Neurosci. Methods 259, 13–21. 10.1016/j.jneumeth.2015.11.019 26658223

