# Supplementary material for: Estimation of Synaptic Conductances in Presence of Nonlinear Effects Caused by Subthreshold Ionic Currents
Source: Front Comput Neurosci. 2017 Jul 25;11:69. doi: 10.3389/fncom.2017.00069 (PMC5524927; doi:10.3389/fncom.2017.00069)
Supplement: Supplementary file 1 [file DataSheet1.pdf]

Appendix: Estimation of synaptic conductances in presence of nonlinear effects caused by subthreshold ionic currents

C. Vich [1,*], R. W. Berg [2], A. Guillamon [3] and S. Ditlevsen [4]

*Correspondence:
C. Vich, Departament de Matemàtiques i Informàtica, Universitat de les Illes Balears, Cra. Valldemossa Km. 7,5, 07122 Palma, Spain
catalina.vich@uib.es

APPENDIX A MATHEMATICAL MODEL OF THE STELLATE NEURON

The stellate model is taken from Rotstein et al. (2006), where the membrane potential dynamics is given by

$$C\frac{dV}{dt} = -I_L - I_{Na} - I_K - I_{NaP} - I_h + I_{app} - I_{syn},$$

where C is the membrane capacitance, I_{syn} the synaptic current and I_{app} the applied current. The leakage and ion currents are given by:

$$\begin{aligned}
&I_L = g_L(v - V_L),\\
&I_{Na} = g_{Na}m^3(v)h(v - V_{Na}),\\
&I_K = g_K n^4(v - V_K),\\
&I_{NaP} = g_p p(v - V_{Na}),\\
&I_h = g_h(0.65r_f + 0.35r_s)(v - V_h),
\end{aligned}$$

where V_{ion} and g_{ion} denote the corresponding ion reversal potential and maximal conductance, respectively.

The gating variables w, which can be either $m,\ h,\ n,\ p,\ r_f$ or r_s, follow the differential equation

$$\frac{dw}{dt} = \frac{w_\infty(v) - w}{\tau_w(v)}$$

where

$$w_\infty(v) = \frac{\alpha_w(v)}{\alpha_w(v) + \beta_w(v)}, \qquad \tau_w(v) = \frac{1}{\alpha_w(v) + \beta_w(v)},$$

and the gating dynamics are given by:

$$\begin{aligned}
&\alpha_m(v) = -0.1\,(v+23)/(\exp(-0.1(v+23))-1),\\
&\beta_m(v) = 4\,\exp(-(v+48)/18),\\
&\alpha_h(v) = 0.07\,\exp(-(v+37)/20),\\
&\beta_h(v) = 1/(1+\exp(-0.1(v+7))),\\
&\alpha_n(v) = -0.01\,(v+27)/(\exp(-0.1(v+27))-1),\\
&\beta_n(v) = 0.125\,\exp(-(v+37)/80),\\
&\alpha_p(v) = 1/(0.15(1+\exp(-(v+38)/6.5))),\\
&\beta_p(v) = \exp(-(v+38)/6.5)/(0.15(1+\exp(-(v+38)/6.5))),\\
&r_{f,\infty}(v) = 1/(1+\exp((v+79.2)/9.78)),\\
&\tau_{r_f}(v) = 0.51/(\exp(v-1.7)/10+\exp(-(v+340)/52))+1,\\
&r_{s,\infty}(v) = 1/(1+\exp((v+2.83)/15.9))^{58},\\
&\tau_{r_f}(v) = 5.6/(\exp(v-1.7)/14+\exp(-(v+260)/43))+1.
\end{aligned}$$

The biophysical parameters are:

Conductances $(mS/cm^2): g_L = 0.1,\ g_{Na} = 52,\ g_K = 11,\ g_p = 0.5,\ g_h = 1.5,$
Reversal potentials $(mV): V_L = -65,\ V_{Na} = 55,\ V_K = -90,\ V_h = 0.20,\ V_E = 0,\ V_I = -80;$
Capacitance $(\mu F/cm^2): C = 1.$

In order to avoid spikes in the model dynamics, a current of $-7.2\mu A/cm^2$ has been applied.

APPENDIX B MAXIMUM LIKELIHOOD METHOD

The Euler discretization of equation (2) locally follows a Gaussian distribution with mean and variance $V_n + (aV_n^2 + bV_n + c)\Delta$ and $\sigma^2\Delta$, respectively. Hence, the conditional probability density function is given by

$$p(V_n|V_{n-1}) = \frac{1}{\sqrt{2\pi\sigma^2\Delta}} \exp\left\{-\frac{\left(V_n - V_{n-1} - (aV_{n-1}^2 + bV_{n-1} + c)\Delta\right)^2}{2\sigma^2\Delta}\right\},$$

and consequently, the log-likelihood function is

$$\log \mathcal{L}(\theta) = \sum_{n=1}^{M} \left[\log\left(\frac{1}{\sqrt{2\pi\sigma^2\Delta}}\right) - \frac{\left(V_n - V_{n-1} - (aV_{n-1}^2 + bV_{n-1} + c)\Delta\right)^2}{2\sigma^2\Delta}\right]$$

where θ is the vector of unknown parameters.

We consider two different cases of unknown parameters: when both α and the conductances are unknown such that $\theta = (a, b, c)^T$; and finally, when only the conductances are supposed unknown such that $\theta = (b, c)^T$. Next we compute the maximum of the log-likelihood in each situation.

- When $\theta = (a, b, c)^T$, the maximum of the log-likelihood function is computed by solving the system

$$\left\{\frac{\partial \log \mathcal{L}(\theta)}{\partial a} = 0, \quad \frac{\partial \log \mathcal{L}(\theta)}{\partial b} = 0, \quad \frac{\partial \log \mathcal{L}(\theta)}{\partial c} = 0\right\}.$$

The solution, providing estimators of parameters a, b and c, satisfies the following linear system:

$$\begin{pmatrix} \sum_{n=1}^{M} V_{n-1}^4\Delta & \sum_{n=1}^{M} V_{n-1}^3\Delta & \sum_{n=1}^{M} V_{n-1}^2\Delta \\ \sum_{n=1}^{M} V_{n-1}^3\Delta & \sum_{n=1}^{M} V_{n-1}^2\Delta & \sum_{n=1}^{M} V_{n-1}\Delta \\ \sum_{n=1}^{M} V_{n-1}^2\Delta & \sum_{n=1}^{M} V_{n-1}\Delta & \sum_{n=1}^{M} \Delta \end{pmatrix} \cdot \begin{pmatrix} a \\ b \\ c \end{pmatrix} = \begin{pmatrix} \sum_{n=1}^{M} (V_n V_{n-1}^2 - V_{n-1}^3) \\ \sum_{n=1}^{M} (V_n V_{n-1} - V_{n-1}^2) \\ \sum_{n=1}^{M} (V_n - V_{n-1}) \end{pmatrix}. \tag{S1}$$

- Finally, when $\theta = (b, c)^T$ the maximum of the log-likelihood function is computed by solving the system
$$\left\{ \frac{\partial \log \mathcal{L}(\theta)}{\partial b} = 0, \quad \frac{\partial \log \mathcal{L}(\theta)}{\partial c} = 0 \right\},$$
obtaining estimators of parameters b and c as solutions of the linear system

$$\begin{pmatrix} \sum_{n=1}^{M} V_{n-1}^2\Delta & \sum_{n=1}^{M} V_{n-1}\Delta \\ \sum_{n=1}^{M} V_{n-1}\Delta & \sum_{n=1}^{M} \Delta \end{pmatrix} \cdot \begin{pmatrix} b \\ c \end{pmatrix} = \begin{pmatrix} \sum_{n=1}^{M} (V_n V_{n-1} - V_{n-1}^2) - a\sum_{n=1}^{M} V_{n-1}^3\Delta \\ \sum_{n=1}^{M} (V_n - V_{n-1}) - a\sum_{n=1}^{M} V_{n-1}^2\Delta \end{pmatrix} \tag{S2}$$

REFERENCES

Rotstein, H., Oppermann, T., White, J., Kopell, N., 2006. The dynamic structure underlying subthreshold oscillatory activity and the onset of spikes in a model of medial entorhinal cortex stellate cells. Journal of Computational Neuroscience 21 (3), 271–292.
URL `http://dx.doi.org/10.1007/s10827-006-8096-8`
