# Supplementary material for: Estimation of Synaptic Conductances in Presence of Nonlinear Effects Caused by Subthreshold Ionic Currents
Source: Front Comput Neurosci. 2017 Jul 25;11:69. doi: 10.3389/fncom.2017.00069 (PMC5524927; doi:10.3389/fncom.2017.00069)
Supplement: Supplementary file 2 [file Presentation1.pdf]

Supplementary Material:
Estimation of synaptic conductances in presence of nonlinear effects caused by subthreshold ionic currents

C. Vich [1,*], R. W. Berg [2], A. Guillamon [3] and S. Ditlevsen [4]
*Correspondence:
C. Vich, Departament de Matemàtiques i Informàtica, Universitat de les Illes Balears, Cra. Valldemossa Km. 7,5, 07122 Palma, Spain
catalina.vich@uib.es

In this supplementary material we present some results obtained using the QIF method (Algorithm 1 in the article) when data are generated from different neuron models. These models are: the QIF model, the stellate model and a modified pyramidal model. The first two models are presented in the article (see Sections 2.4.1 and 2.4.2 in the article). The last model, which is not presented in the article, consists, briefly explained, of a modification of the pyramidal cell model described in Wang (1998) where we only consider the equation for the soma of the neuron, and we add a low-threshold current to have a variety of non-linear subthreshold currents, see Appendix A for more details on the model.

In Section 1 we plot the results obtained by the QIF method when different *in silico* data traces are obtained. Section 1.1 depicts the results obtained when data are generated by the QIF model. These results are just different representations of the results obtained in the manuscript (Figure 2), which might clarify, visually, the goodness of the approximation. In Section 1.2 we depict the results obtained when data are generated by the stellate neuron model. The conductances are estimated by the QIF method with a smaller sliding window ($50\ ms$ instead of $100\ ms$). Finally, in Section 1.3, we present the results obtained when data are generated from a modified pyramidal model. We compare results to those obtained with the OU and the oversampling methods (see Section 2.2 and 2.3 in the article for more details about these methods).

In Section 2, we compute the mean squared errors (MSE) obtained using data from the pyramidal model (Section 2.1), and we study the robustness of the QIF method under different realizations (Section 2.2).

1 SUPPLEMENTARY FIGURES

In this section we present results obtained when applying the QIF method to *in silico* data obtained from different neural models: the QIF model, the stellate cell model, and the modified pyramidal cell model.

1.1 Results for the QIF model

Results for the QIF neuron model (see also Section 2.4.1 in the article) are depicted in Figure S1. The true excitatory and inhibitory traces are well approximated by the estimated traces. However, Panels A and B show that the inhibitory trace is estimated much less accurate than the excitatory one. This is also seen in panels D and E, where dots lie closer to the identity line for the excitatory than for the inhibitory conductances.

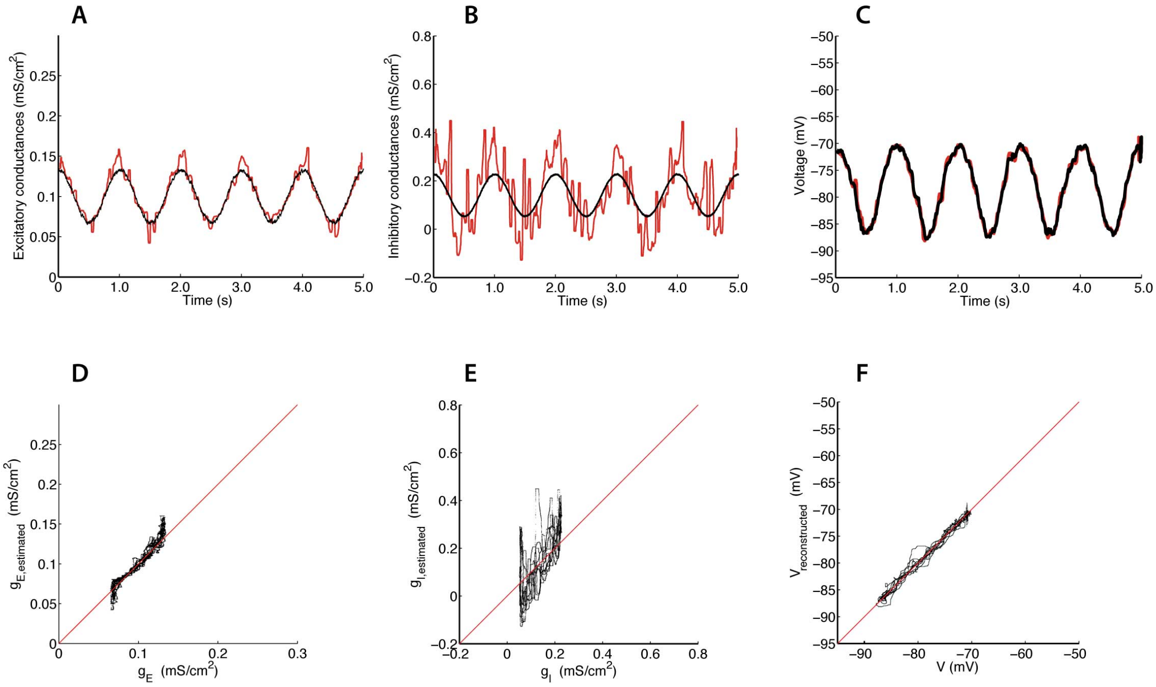

Figure S1. Estimation by the QIF method of the conductances from data generated from the QIF neuron model. A: true (black) and estimated (red) excitatory conductances. B: true (black) and estimated (red) inhibitory conductances. C: simulated voltage using the true conductances (black) and the estimated conductances (red). D, E, F: scatter plots of the estimated versus the true excitatory conductances (D), inhibitory conductances (E), and voltage (F); the red lines are the identity lines. The data have been obtained each $dt = 0.05\ ms$ and the MLE is applied with a $l = 50\ ms$ sliding window. The neuron parameters are given in Section 2.4.1 in the article, and the synaptic drive description is given in Section 2.4.4 in the article. Estimation has been performed using the QIF method given in Algorithm 1 in the article.

1.2 Results for the stellate model

Results for the stellate neuron model (see also Section 2.4.2 in the article) are depicted in Figure S2. Contrary to the article, in this figure we have used a smaller sliding window of $50\ ms$ in the QIF method. Although this window size causes higher MSEs in the estimation (see Table 2 in the article), both the excitatory and the inhibitory traces are well captured.

1.3 Results for the pyramidal model

Results for the modified pyramidal models are depicted in Figure S3. The true excitatory trace is well approximated by the estimated trace, dots in panel D are close to the identity line, whereas the estimation of the inhibitory conductances only captures the overall shape and level, and is much more noisy, where dots in panel E are not as close to the identity line, even though they are located in the vicinity.

In Figure S4 the results obtained with the QIF method are compared to the OU and the oversampling method. Panels A and B depict the time courses of the true conductances and the estimates from the three methods, and panels C and D show scatter plots of the estimated conductances versus the prescribed ones. Both the QIF and the OU methods concentrate around the identity line, indicating acceptable fits. The QIF method provides a slight improvement on the estimated excitatory conductance compared to the OU

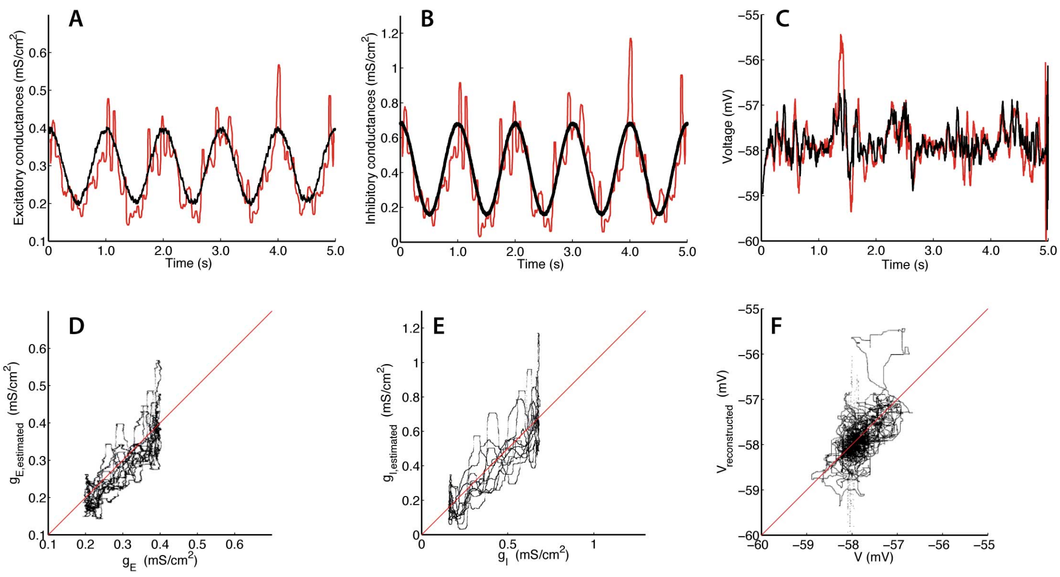

Figure S2. Estimation by the QIF method of conductances from data generated from the stellate cell model. A: true (black) and estimated (red) excitatory conductances. B: true (black) and estimated (red) inhibitory conductances. C: simulated voltage using the true conductances (black) and the estimated conductances (red). D, E, F: scatter plots of the estimated versus the true excitatory conductances (D), inhibitory conductances (E), and voltage (F); the red lines are the identity lines. The data were generated each $dt = 0.05\ ms$ and the MLE sample window was $l = 50\ ms$. The neuron parameters are given in Section 2.4.2 and the synaptic drive description is given in Section 2.4.3 in the article. For this model, we have tripled the conductance traces obtained from equation (8) in the article in order to induce higher subthreshold activity. Estimation has been performed using the QIF method given in Algorithm 1 in the article.

method, whereas the inhibitory conductance is considerable better estimated with the QIF compared to the OU method. The oversampling method fails and should not be used for this type of data.

2 SUPLEMENTARY TABLES

In this section we present different tables showing the mean squared errors (MSEs) obtained, first, when the QIF method is applied to the modified pyramidal cell model; and, second, when it is applied to both the stellate and the pyramidal models, but using different random seeds.

2.1 Goodness of the results of the pyramidal model

To quantify and compare the errors of the different estimation procedures applied to *in silico* data generated by the modified pyramidal model, the mean squared errors (MSEs) and bias are shown in Table S1. The table is consistent with Figure S4 and also with results obtained from data generated by the stellate model (see Section 3.3 in the article). However, the bias is now slightly smaller for the OU method.

2.2 Robustness of the QIF method under different realizations

Table S2 and S3 depict the MSEs obtained when the QIF and the OU methods have been applied to different data traces generated using the stellate and the modified pyramidal model, respectively. These traces have been generated by repeating the simulation of the model with different random seeds. Both tables show that the errors are qualitatively the same.

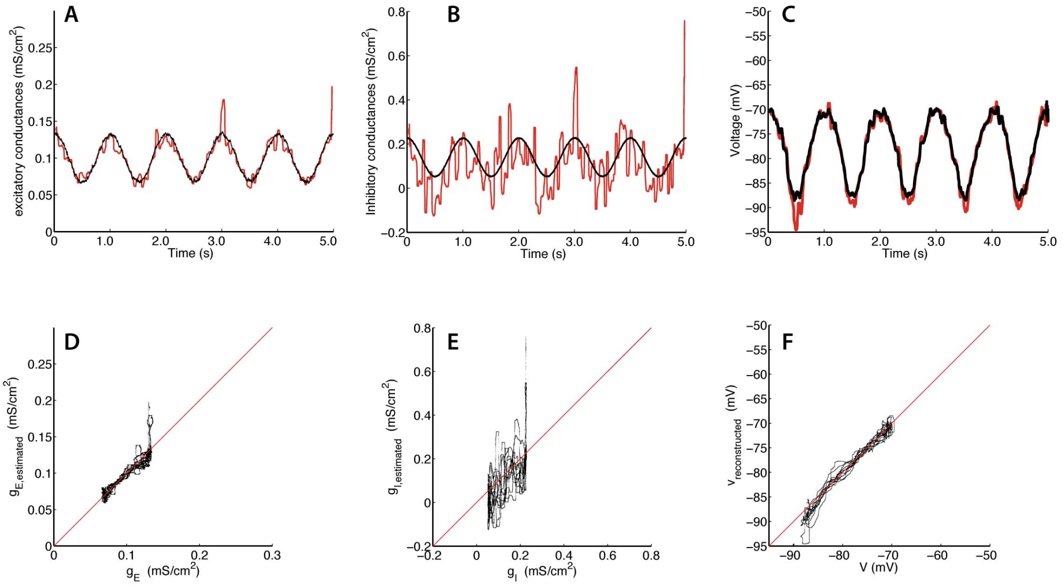

Figure S3. Estimation by the QIF method of conductances from data generated from the pyramidal cell model. A: true (black) and estimated (red) excitatory conductances. B: true (black) and estimated (red) inhibitory conductances. C: simulated voltage using the true conductances (black) and the estimated conductances (red). D, E, F: scatter plots of the estimated versus the true excitatory conductances (D), inhibitory conductances (E), and voltage (F); the red lines are the identity lines. The data were generated with $dt = 0.05\ ms$ and the MLE sample window was $l = 50\ ms$. The neuron parameters are given in Appendix A, and the synaptic drive description is given in Section 2.4.4 in the article. Estimation has been performed using the QIF method given in Algorithm 1 in the article.

Estimation Procedure	g_E MSE	g_I MSE	g_E bias	g_I bias
QIF method	$7.42 \cdot 10^{-5}$	$9.51 \cdot 10^{-3}$	$2.41 \cdot 10^{-3}$	$1.02 \cdot 10^{-1}$
OU method	$3.67 \cdot 10^{-4}$	$3.97 \cdot 10^{-2}$	$1.01 \cdot 10^{-3}$	$-7.05 \cdot 10^{-2}$
Oversampling method	$1.96 \cdot 10^{-2}$	$4.61 \cdot 10^{-1}$	$6.82 \cdot 10^{-2}$	$1.43 \cdot 10^{-1}$

Table S1. Mean squared errors and bias of the different estimation procedures when applied to the modified pyramidal cell model. Data have been generated from the modified pyramidal cell model described in Appendix A with synaptic drive given in Section 2.4.4. The window size used in the QIF and the OU methods is $l = 50\ ms$. In the oversampling method, parameter values are $\kappa_\alpha = \kappa_\beta = 0.5$. For the QIF method, the estimated conductances have been filtered by the median filter using a sample window of $50\ ms$. The MSE are given for excitatory conductances (second column) and inhibitory conductances (third column). Bias is given for excitatory conductances (third column) and inhibitory conductances (fourth column).

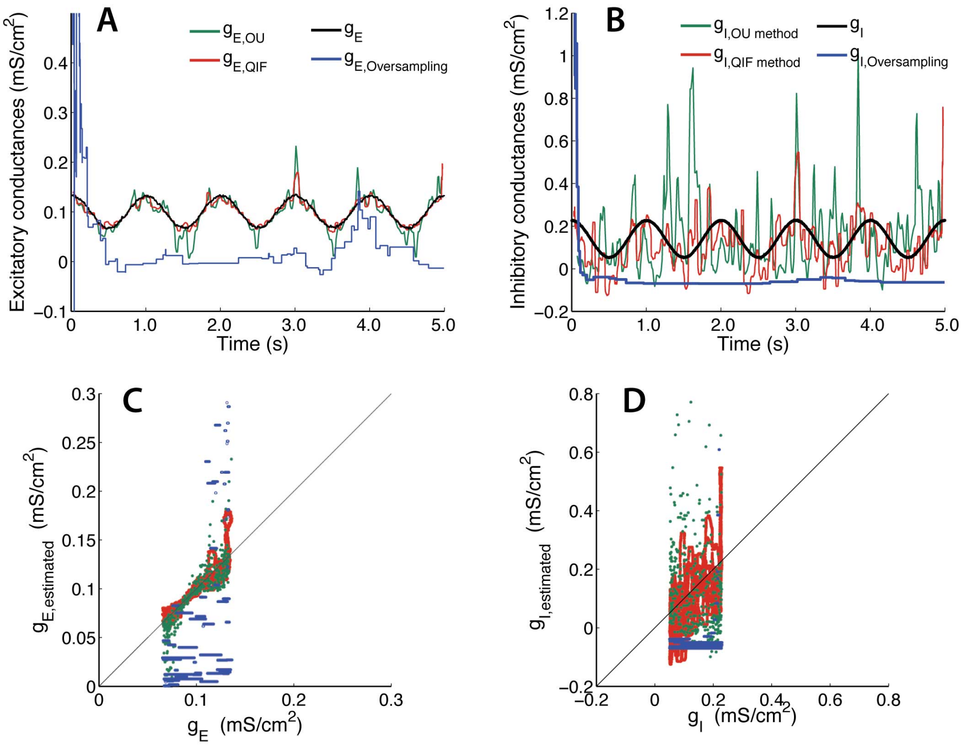

Figure S4. Comparison between single-trial estimation procedures; QIF, OU and oversampling method on data generated from the pyramidal model. Panels A and B show the prescribed and the estimated synaptic input generated from equation (8) in the article. A: Excitatory conductance. B: Inhibitory conductance. Panels C and D show scatter plots of the estimated conductances versus the true ones. C: Excitatory conductances. D: Inhibitory conductances. The sample window used in the QIF and the OU methods is $l = 50\ ms$. In the oversampling method, parameter values are $\kappa_\alpha = \kappa_\beta = 0.5$. For the QIF method, the estimated conductances have been filtered by the median filter using a sample window of $50\ ms$.

	QIF method		OU method	
Simulation Num.	g_E	g_I	g_E	g_I
1	$2.03 \cdot 10^{-3}$	$9.44 \cdot 10^{-3}$	$1.13 \cdot 10^{-2}$	$1.15 \cdot 10^{-1}$
2	$2.60 \cdot 10^{-3}$	$1.54 \cdot 10^{-2}$	$9.67 \cdot 10^{-3}$	$1.16 \cdot 10^{-1}$
3	$2.07 \cdot 10^{-3}$	$1.14 \cdot 10^{-2}$	$6.96 \cdot 10^{-3}$	$1.25 \cdot 10^{-1}$
4	$2.23 \cdot 10^{-3}$	$1.34 \cdot 10^{-2}$	$1.17 \cdot 10^{-2}$	$1.19 \cdot 10^{-1}$
5	$1.96 \cdot 10^{-3}$	$1.17 \cdot 10^{-2}$	$8.86 \cdot 10^{-3}$	$1.26 \cdot 10^{-1}$
6	$1.90 \cdot 10^{-3}$	$1.15 \cdot 10^{-2}$	$1.05 \cdot 10^{-2}$	$1.34 \cdot 10^{-1}$

Table S2. Mean squared errors of the excitatory and inhibitory conductances repeating the simulation with different random seeds using data generated from the stellate model. We present the MSE given when both the QIF and the OU methods have been applied to a data trace generated by the pyramidal neuron model described in Section 2.4.2 in the article with synaptic drive given in Section 2.4.4 in the article. The window size used is $l = 50\ ms$ in both cases. The estimated conductances have been filtered by the median filter also using a sample window of $50\ ms$.

	QIF method		OU method	
Simulation Num.	g_E	g_I	g_E	g_I
1	$7.42 \cdot 10^{-5}$	$9.51 \cdot 10^{-3}$	$3.67 \cdot 10^{-4}$	$3.97 \cdot 10^{-2}$
2	$4.86 \cdot 10^{-5}$	$8.16 \cdot 10^{-3}$	$5.95 \cdot 10^{-4}$	$9.33 \cdot 10^{-2}$
3	$5.05 \cdot 10^{-5}$	$7.49 \cdot 10^{-3}$	$4.39 \cdot 10^{-4}$	$7.11 \cdot 10^{-2}$
4	$7.62 \cdot 10^{-5}$	$9.89 \cdot 10^{-3}$	$5.88 \cdot 10^{-4}$	$1.18 \cdot 10^{-1}$
5	$5.73 \cdot 10^{-5}$	$8.91 \cdot 10^{-3}$	$4.98 \cdot 10^{-4}$	$1.04 \cdot 10^{-1}$
6	$6.09 \cdot 10^{-5}$	$1.17 \cdot 10^{-2}$	$4.55 \cdot 10^{-4}$	$9.59 \cdot 10^{-2}$

Table S3. Mean squared errors of the excitatory and inhibitory conductances repeating the simulation with different random seeds using data generated from the pyramidal model. We present the MSE given when both the QIF and the OU methods have been applied to a data trace generated by the pyramidal neuron model described in Appendix A with synaptic drive given in Section 2.4.4 in the article. The window size used is $l = 100\ ms$ in both cases. The estimated conductances have been filtered by the median filter also using a sample window of $50\ ms$.

APPENDIX A MATHEMATICAL MODEL OF THE MODIFIED PYRAMIDAL NEURON

The modified pyramidal model is a version of the model in Wang (1998). Only the axonal initial segment is considered and we add a low-threshold calcium current. The membrane potential is given by

$$C\frac{dV}{dt} = -I_L - I_{Na} - I_K - I_{Ca} - I_{AHP} - I_{LTS} + I_{app} - I_{syn},$$

where C is the capacitance, I_{syn} the synaptic current, and I_{app} the applied current. The leakage and ion currents are given by:

$$\begin{aligned}
I_L &= g_L(v - V_L),\\
I_{Na} &= g_{Na}m_\infty^3(v)h(v - V_{Na}),\\
I_K &= g_K n^4(v - V_K),\\
I_{Ca} &= g_{Ca} ml_\infty(v - V_{Ca}),\\
I_{AHP} &= g_{AHP}\tfrac{c}{c+K_D}(v - V_K),\\
I_{LTS} &= g_{LTS}m_{LTS,\infty}^3 h_{LTS}(v - V_{Ca}),
\end{aligned}$$

where V_{ion} and g_{ion} denote the specific ion reversal potential and maximal conductance, respectively, c is the intracellular calcium concentration, $[Ca^{2+}]$, and K_D denotes a growth factor of the I_{AHP} current. The variables h and n are gating variables governed by first-order kinetics of type

$$\dot{w} = \frac{dw}{dt} = \phi[\alpha_w(v)(1 - w) - \beta_w(v)w] = \phi\frac{w_\infty(v) - w}{\tau_w(v)} \tag{S1}$$

for $w = h$ or n. The m-type variables are assumed to reach their steady-state for a given v instantaneously, that is $m = m_\infty(v)$ and $ml = ml_\infty(v)$. The remaining functions describing the gating dynamics are given

by:

$$
\begin{aligned}
w_\infty(v) &= \alpha_w(v)/(\alpha_w(v)+\beta_w(v)),\\
\tau_w(v) &= 1/(\alpha_w(v)+\beta_w(v)),\\
\alpha_h(v) &= 0.07\ \exp(-(v+50)/10),\\
\beta_h(v) &= 1/(1+\exp(-0.1(v+20))),\\
\alpha_n(v) &= -0.01\,(v+34)/(\exp(-0.1(v+34))-1),\\
\beta_n(v) &= 0.125\ \exp(-(v+44)/25),\\
\alpha_m(v) &= -0.1\,(v+33)/(\exp(-0.1(v+33))-1),\\
\beta_m(v) &= 4\ \exp(-(v+58)/12),\\
ml_\infty(v) &= 1/(1+\exp(-(v+20)/5)),\\
m_{LTS,\infty}(v) &= 1/(1+\exp(-(v+65)/7.8)),\\
dh_{LTS}/dt &= (\phi_{LTS}(h_{LTS,\infty}(v)-h_{LTS}(v)))/\tau_{LTS}(v),
\end{aligned}
$$

where

$$
\begin{aligned}
h_{LTS,\infty}(v) &= 1/(1+\exp((v+81)/11)),\\
\tau_{LTS}(v) &= h_{LTS,\infty}(v)\exp((v+162.3)/17.8).
\end{aligned}
$$

The intracellular calcium concentration $c=[Ca^{2+}]$ is assumed to be governed by a leaky-integrator

$$dc/dt = -\alpha I_{Ca} - c/\tau_{Ca}, \tag{S2}$$

where τ_{Ca} is the time constant and α is proportional to the membrane area divided by the volume below the membrane.

This model is a modification of the pyramidal cell model described in Wang (1998) where we only consider the equation for the soma of the neuron, and we add a low-threshold current to have a variety of non-linear subthreshold currents. The model describes the dynamics of a modified pyramidal neuron with intrinsic currents involving five different channels: the standard sodium, potassium and calcium channels, plus an afterhyperpolarizing calcium-activated potassium (AHP) channel and a T-type calcium channel inducing low-threshold spike (LTS) currents. The AHP-current is activated immediately after the spikes causing subthreshold activity to last around 80 ms after the spike. The LTS-current (Destexhe et al., 1993) is activated in the subthreshold regime, independently of the spiking activity, causing non-linear subthreshold effects.

The biophysical parameters are:

$$
\begin{aligned}
&\text{Conductances}\,(mS/cm^2): g_L=0.1,\ g_{Na}=45,\ g_K=18,\ g_{Ca}=1.0,\ g_{AHP}=5.0,\ g_{LTS}=0.5;\\
&\text{Reversal potentials}\,(mV): V_L=-65,\ V_{Na}=55,\ V_K=-80,\ V_{Ca}=120.0,\ V_E=0,\ V_I=-80;\\
&\text{Capacitance}\,(\mu F/cm^2): C=1;\\
&\text{Non}-\text{dimensional constants}: \phi=4,\ \phi_{LTS}=2;\\
&\text{Other constants}: \alpha=0.002\,\mu M(ms\,\mu A)^{-1}cm^2,\ \tau_{Ca}=80\,ms,\ K_D=30.0\,\mu M.
\end{aligned}
$$

The rest of neuron parameters are set to $V_T = -74.27\ mV$, $I_T = -1.359\ \mu A/cm^2$, and $I_{app} = -8.7\ \mu A/cm^2$. This last value is chosen to be close to the largest current that with high probability do not cause spikes in the model for the given level of noise.

REFERENCES

Wang, X. J., Mar. 1998. Calcium coding and adaptive temporal computation in cortical pyramidal neurons. J Neurophysiol 79 (3), 1549–1566.
URL `http://view.ncbi.nlm.nih.gov/pubmed/9497431`

Destexhe, A., Babloyantz, A., Sejnowski, T., October 1993. Ionic mechanisms for intrinsic slow oscillations in thalamic relay neurons. Biophysical Journal 65, 1538–1552.
URL `http://www.ncbi.nlm.nih.gov/pmc/articles/PMC1225880/`
